# Executive summary of the American Radium Society appropriate use criteria for brain metastases in epidermal growth factor receptor mutated-mutated and ALK-fusion non-small cell lung cancer

**DOI:** 10.1093/neuonc/noae041

**Published:** 2024-03-09

**Authors:** Seema Nagpal, Michael T Milano, Veronica L Chiang, Scott G Soltys, Alexandria Brackett, Lia M Halasz, Amit K Garg, Arjun Sahgal, Manmeet S Ahluwalia, Martin C Tom, Joshua D Palmer, Jonathan P S Knisely, Samuel T Chao, Melanie Hayden Gephart, Tony J C Wang, Simon S Lo, Eric L Chang

**Affiliations:** Department of Neurology, Stanford University, Palo Alto, California, USA; Department of Radiation Oncology, University of Rochester, Rochester, New York, USA; Department of Neurosurgery, Yale University, New Haven, Connecticut, USA; Department of Radiation Oncology, Stanford University, Palo Alto, California, USA; Cushing/Whitney Medical Library, Yale University, New Haven, Connecticut, USA; Department of Radiation Oncology, University of Washington, Seattle, Washington, USA; Department of Radiation Oncology, Presbyterian Healthcare Services, Albuquerque, New Mexico , USA; Department of Radiation Oncology, Sunnybrook Research Institute, Toronto, Ontario, Canada; Department of Medical Oncology, Miami Cancer Institute, Miami, Florida, USA; Department of Radiation Oncology, MD Anderson, Houston, Texas, USA; Department of Radiation Oncology, Ohio State University, Colombus, Ohio, USA; Department of Radiation Oncology, Weill Cornell Medicine, New York, New York, USA; Department of Radiation Oncology, Case Western University, Cleveland, Ohio, USA; Department of Neurosurgery, Stanford University, Palo Alto, California, USA; Department of Radiation Oncology, Columbia University, New York, New York, USA; Department of Radiation Oncology, University of Washington, Seattle, Washington, USA; Department of Radiation Oncology, University of Southern California, Los Angeles, California, USA

**Keywords:** anaplastic lymphoma kinase (ALK), brain metastases, epidermal growth factor receptor (EGFR), non-small cell lung cancer (NSCLC), radiotherapy

## Abstract

The American Radium Society (ARS) Central Nervous System (CNS) committee reviewed literature on epidermal growth factor receptor mutated (EGFRm) and ALK-fusion (ALK+) tyrosine kinase inhibitors (TKIs) for the treatment of brain metastases (BrMs) from non-small cell lung cancers (NSCLC) to generate appropriate use guidelines addressing use of TKIs in conjunction with or in lieu of radiotherapy (RT). The panel developed three key questions to guide systematic review: can radiotherapy be deferred in patients receiving EGFR or ALK TKIs at (1) diagnosis or (2) recurrence? Should TKI be administered concurrently with RT (3)? Two literature searches were performed (May 2019 and December 2023). The panel developed 8 model cases and voted on treatment options using a 9-point scale, with 1–3, 4–6 and 7–9 corresponding to usually not appropriate, may be appropriate, and usually appropriate (respectively), per the UCLA/RAND Appropriateness Method. Consensus was achieved in only 4 treatment scenarios, all consistent with existing ARS-AUC guidelines for multiple BrM. The panel did not reach consensus that RT can be appropriately deferred in patients with BrM receiving CNS penetrant ALK or EGFR TKIs, though median scores indicated deferral may be appropriate under most circumstances. Whole brain RT with concurrent TKI generated broad disagreement except in cases with 2–4 BrM, where it was considered usually not appropriate. We identified no definitive studies dictating optimal sequencing of TKIs and RT for EGFRm and ALK+ BrM. Until such studies are completed, the committee hopes these cases guide decision- making in this complex clinical space.

Brain metastases (BrM) from non-small-cell lung cancer (NSCLC) are common and potentially morbid, occurring in 15%–20% of patients at the time of diagnosis^1,2^ and up to 40% at some point in their disease course.^3^ Depending on the size and location of BrM, patients can develop focal neurologic symptoms, headaches, or seizures. Leptomeningeal metastases (LM), which can cause highly morbid cranial nerve deficits and life-threatening elevated intracranial pressures, occur in ~1%–5% of patients with solid tumors, though autopsy series suggest the incidence in patients with neurologic signs or symptoms may be closer to 10%.^4,5^ Patients with NSCLC harboring certain targetable genetic alterations, such as epidermal growth factor receptor mutations (EGFRm) or anaplastic lymphoma kinase fusions (ALK+), may be significantly more susceptible to developing BrM and LM; some studies suggest this risk could be as much as 2-fold higher compared to patients with EGFR wild-type NSCLC.^6–8^ The 3-year risk of LM in EGFRm NSCLC has been reported to be as high as 25%.^9^

The treatment paradigm for BrM has long relied on surgery and radiotherapy. Historically, systemic chemotherapy was not recommended for treatment of BrM, presumably due to difficulty penetrating the blood-brain barrier, resulting in low intracranial response rates, from <5% to 35%.^10–13^ As radiotherapy and surgical techniques have evolved, there has been increasing utilization of stereotactic radiosurgery (SRS), while reserving whole brain radiation (WBRT) for patients with larger numbers of metastases or LM, leading to relatively clear surgical and radiotherapy guidelines from multiple groups, such as the American Society for Radiation Oncology, the American Radium Society (ARS), and the Congress of Neurological Surgeons.^14–16^ However, in this period, new central nervous system (CNS) penetrant systemic therapies, including targeted tyrosine kinase inhibitors (TKI), immunotherapies, and antibody-drug conjugates, have become widely available. These disease-specific targeted drugs add complexity when formulating universal treatment algorithms. This is particularly relevant in patients with NSCLC, where EGFR mutations and ALK gene fusions are found in approximately 30% and 4%–6% of patients, respectively.^17–19^ Gefitinib, an EGFR TKI, was granted an accelerated FDA approval in 2003, for treatment of locally advanced or metastatic NSCLC refractory to platinum and docetaxel. Approval of erlotinib followed shortly thereafter, in 2004. While patients with CNS disease were excluded from the definitive gefitinib and erlotinib studies, the first case reports demonstrating CNS activity in patients with BrM were published within a year of gefitinib’s approval.^20,21^ Many TKIs have molecular weights in the 400 g/mol range, which along with other factors, such as lipophilicity, may increase blood-brain-barrier penetration, resulting in improved intracranial control.^22^ Early case series of EGFR TKIs suggested intracranial response rates as high as 80%,^23^ leading some physicians to consider deferring CNS radiotherapy in select patients.

In the 2 decades following the first EGFR TKI’s approval, there have been 5 EGFR-specific TKIs and 5 ALK TKIs approved by the FDA. Hundreds of retrospective series that address ALK and EGFR TKI activity in BrM have been published in this timeframe, but the number of well-designed prospective studies addressing patients with BrM is limited. Patients with BrM or LM are often explicitly excluded from novel drug studies. When included, the data are complicated by lack of uniform measures for diagnosing and tracking disease. How and when these drugs are used to treat patients with CNS disease varies widely, often depending on the customs of the physician’s institution or primary specialty. To address this gap, the ARS committee for CNS malignancies systematically reviewed the literature on EGFR and ALK TKIs for the treatment of BrM to generate practical guidelines specifically addressing the use of TKIs in conjunction with, or in lieu of, radiotherapy. Though EGFRm and ALK + disease represent a fraction of NSCLCs, the number of druggable oncologic targets is expanding rapidly. Targets like KRAS may allow patients with multiple primary cancer types to be treated with an oral TKI. The questions raised about radiotherapy and TKI timing in the EGFRm and ALK + populations are, therefore, increasingly relevant to the broader oncology community.

## Materials and Methods

The ARS CNS malignancies panel includes 10 radiation oncologists, 2 neurosurgeons, and 2 neuro-oncologists. Two panel members identify as community radiation oncologists while the remainder are associated with academic medical centers. The panel developed 3 key questions (KQs) to guide the literature search.

**KQ1**: Can radiation therapy (SRS or WBRT) be deferred in patients receiving a potentially CNS-active TKI?
**KQ2**: If radiation therapy is recommended (SRS or WBRT), should it be given concurrently with TKI or in sequence?
**KQ3**: At the time of CNS progression on TKI, should radiation therapy be deferred if another CNS-penetrating TKI is available?

### Literature Search

An initial search of medical literature from peer-reviewed journals was conducted on May 10, 2019 (no preassigned earliest date) using the preferred reporting items for systematic reviews and meta-analyses (PRISMA) guidelines^24^ to search the OVID Medline, OVID Embase, Web of Science, PubMed, and Cochrane databases to retrieve a comprehensive set of relevant articles. The search strategy was developed based on National Library of Medicine® Medical Subject Headings (MeSH®) with the addition of subject-specific keywords. The bibliographies of full articles were reviewed to exclude studies which were not relevant. The literature was reviewed for quality of study design, cohort size, selection bias, evaluation of participants in relation to time from exposure, and methods of assessments. A well-established methodology, the RAND/UCLA modified Delphi, was used by the expert panel to rate the appropriate use of procedures.^25,26^

The search identified 5035 references, with 738 remaining after excluding articles on unrelated topics, duplicates, case reports, animal studies, immunotherapy, drug development reports, pediatric articles, reviews, and those without mention of brain metastases. After initial screening, 117 articles were screened in detail by 4-panel members, who identified 40 full-text studies to guide committee voting. These articles were categorized by paired panel members for relevance to KQs and level of evidence. These 40 studies are included in the evidence table (Supplementary Table A). During article review, case formulation, and voting rounds, additional relevant studies were published. The committee reviewed these on a rolling basis. To assure that studies with direct, high-level evidence relevant to the KQs had not been overlooked, a second literature search was performed on December 20, 2023, yielding 2952 additional references. These were screened by 3 panelists who identified 16 papers of interest. While the panelists’ voting predated this search, there were no recent prospective, randomized studies that directly addressed the KQs or would have impacted voting.

### Representative Cases and Variants

The committee developed 8 representative cases (C), each with up to 5 variants (CVs), to address the KQs. Variables included the number of BrM, availability of mutational testing information, patient symptoms, and in case 8, variable activity of a hypothetical new TKI. Within each case variant, up to 16 management options were considered, for a total of 150 management options/treatment scenarios. Using a 9-point scale (1–3 *usually not appropriate*, 4–6 *may be appropriate*, 7–9 *usually appropriate*), the panel voted on the appropriateness of each management option. Agreement and disagreement were decided by the number of outliers relative to the number of votes. Per RAND criteria, in a panel of 11–13 voters, agreement requires ≤3 ratings outside of the 3-point region containing the median. Specifically, agreement does *not* require all panelists voting within a single category (1–3, 4–6, or 7–9). For purposes of this manuscript, the authors define this as consensus. Disagreement occurred when ≥4 ratings fell into the extreme ranges (1–3 and 7–9). Prior to each voting rounds 2 and 3, areas of disagreement were discussed and re-voted upon for a total of 3 voting rounds. Panel members could abstain from specific voting scenarios, and some radiation oncology members were unable to participate in all rounds, as these occurred at the height of the COVID-19 pandemic. One member participated in article review and case creation, but did not vote. Each management option was rated as “usually appropriate,” “may be appropriate,” or “usually not appropriate” based on voting. Areas of disagreement defaulted to “may be appropriate.” Results were collated and analyzed to develop summarized appropriate use criteria. Using ARS methodology, located at https://www.americanradiumsociety.org/page/aucmethodology, each recommendation was rated as “strong,” “moderate,” or “conditional,” and the strength of evidence was rated as “strong,” “moderate,” “limited,” “expert consensus,” or “expert opinion.”

## Results

Supplementary Table B shows each C, CV, and the voting breakdown for the specific treatment options identified by the panel. After 3 rounds of voting, there was substantial lack of agreement across most management scenarios.

While ARS methodology does not require consensus to be classified as “agreement,” notable areas of consensus, where all votes fell into a single rating category, occurred in treatment scenarios covered by existing ARS-AUC Guidelines for SRS for multiple brain metastases, independent of TKI availability.^27^ For treatment of newly diagnosed EFGRm or ALK + NSCLC with multiple asymptomatic BrM, SRS to all BrM followed by TKI was *usually appropriate* for patients with 2–4 BrMs (CV1a). Case 3 described the treatment of newly diagnosed EFGRm or ALK + NSCLC with 2 symptomatic, unresectable BrM. In this scenario, SRS for both lesions followed by TKI was *usually appropriate*. Similarly, for patients with symptomatic and surgically accessible BrMs at the time of initial diagnosis of NSCLC (C6), there was clear consensus that surgery, followed by SRS and then TKI was *usually appropriate.* SRS followed by TKI was also considered *usually appropriate* in a case of isolated CNS progression in a patient on third-generation TKI with 7 new BrM (CV5a). In no management scenario was there consensus that TKI could be used in lieu of RT.

*KQ 1: Can radiation therapy (SRS or WBRT) be deferred in patients receiving a potentially CNS-active TKI?*

KQ1 addresses the deferral of radiotherapy with the use of TKI alone for BrM in patients who will receive a CNS-active drug. KQ1 was addressed directly in 5 of the 8 cases, in 7 specific treatment scenarios (Table 1). In treatment-naïve patients, there was no treatment scenario that garnered consensus or agreement that radiotherapy for BrM can be deferred in patients who will receive a CNS penetrant TKI. Under most circumstances, votes clustered in the usual and may be appropriate categories. Thus, per methodology, the recommendation defaults to “may be appropriate” to defer radiotherapy in treatment-naïve patients with BrM from EGFRm or ALK + NSCLC. These recommendations are conditional as supporting evidence is limited to retrospective studies or prospective studies randomized between TKIs, not with radiotherapy.

**Table 1.** Panelist Voting on Treatment Scenarios Addressing KQ1: The Use of TKI Alone, in Lieu of Radiation, in Newly Diagnosed NSCLC With CNS Metastases

Clinical Scenario (Case or Case Variant)	Agreement or Disagreement	Vote Breakdown	Recommendation	SOR	SOE
Newly diagnosed EGFR/ALK NSCLC with 2–10 asymptomatic BrMs (CV1a and b)	Disagreement	11 of the 12 votes for may be or usually appropriate	*May be appropriate*	Conditional	Moderate^28–33^
Newly diagnosed EGFR/ALK NSCLC with 11 to >20 asymptomatic BrMs (CV1c and d)	Disagreement	All 12 votes within may be or usually appropriate	*May be appropriate*	Conditional	Expert opinion^*^
Newly diagnosed EGFR/ALK NSCLC with 2 symptomatic, unresectable BrMs (C3)	Disagreement	All 12 votes within usually not or may be appropriate	*May be appropriate*	Conditional	Expert opinion^*^
Newly diagnosed EGFR/ALK NSCLC with multiple BrMs, 2 of larger size and symptoms at time of diagnosis (C4)	Disagreement	Broadly distributed across 3 categories	*May be appropriate*	Conditional	Expert opinion^*^
Newly diagnosed EGFR/ALK NSCLC with a single symptomatic, surgically resectable BrM (C6)	Disagreement	Clustered in not usually appropriate and may be appropriate	*May be appropriate*	Conditional	Expert opinion^*^
Newly diagnosed EGFR/ALK NSCLC with asymptomatic diffuse LM (CV7a)	Agreement with recommendation based on median rating (7)	All votes are within may be and usually appropriate	*Usually appropriate*	Strong	Moderate^29,30,32,34,35^
Newly diagnosed EGFR/ALK NSCLC with symptomatic diffuse LM (CV7b)	Disagreement	All votes are within may be and usually appropriate	*May be appropriate*	Conditional	Expert opinion^*^

^*^The evidence for this recommendation is based on extrapolations from retrospective data and meta-analysis. These treatment scenarios are not directly covered in an individual manner by specific studies.

In the special circumstance of patients with newly diagnosed asymptomatic, diffuse LM from ALK + or EGFRm NSCLC, the panel agreed that TKI alone was *usually appropriate*. There was, however, disagreement on using TKI alone for patients with symptomatic LM.

### Evidence to Support Decision-Making for KQ1

While our literature review identified multiple phase 3 studies randomizing patients with or without BrM to one TKI versus another, only 1 prospective, randomized trial compared an EGFR TKI directly to radiotherapy. The BRAIN study randomized 176 treatment-naïve patients with ≥3 BrM from EGFRm NSCLC to receive first-generation EGFR inhibitor icotinib, or WBRT (30 Gy in 10 fractions) with concurrent or sequential chemotherapy.^28^ The study’s primary endpoint was intracranial progression-free survival (PFS; IC-PFS) by RECIST criteria. Patients with progression were able to cross treatment arms. The group receiving upfront icotinib had longer IC-PFS than those who received WBRT with chemotherapy (10 v. 4.8 mo., HR = 0.56, *P* = .014), but there was no significant difference in median overall survival (mOS 18 v. 20.5 mo., HR = 0.93, *P* = .734). This study has several limitations, including the intention to use WBRT concurrently with chemotherapy, which may increase AEs without benefit in survival,^36,37^ and a dropout rate of nearly 20% in patients randomized to WBRT. Additionally, icotinib is not approved in the United States. While the development of ALK inhibitors has included more patients with BrM from the outset, our literature review did not identify any prospective studies directly comparing ALK inhibitors to CNS-directed radiotherapy.

The paucity of prospective trials comparing EGFR and ALK TKIs directly to radiotherapy for CNS disease stands in stark contrast to the hundreds of retrospective studies in this space. One of the largest series was reported by Magnuson et al., which compared 351 patients with EGFRm NSCLC BrM across 6 institutions who received upfront first-generation EGFR TKI alone, SRS with adjuvant TKI or WBRT with adjuvant TKI.^38^ The mOS for patients who received upfront SRS, WBRT, and TKI first was 46, 30, and 25 months, respectively (*P* < .001). Patients who received WBRT generally had less favorable prognosis. Patients in the WBRT and TKI groups were more likely to have stage IV disease at initial diagnosis. The authors conclude that deferral of radiotherapy was associated with inferior OS. Due to its size and lack of other randomized data, the committee included this and several other large retrospective series in the evidence table.

Given direct head-to-head data are sparse, committee members frequently referred to studies describing single-agent activity of EGFR and ALK TKIs. Most of these studies were *not* included in the evidence table because they do not directly address the efficacy of radiotherapy or its timing. Additionally, articles that do not include BrM or LM in the title, abstract, or keywords may not reliably appear in database searches. However, these articles provide the clearest comparators to the prospective data for SRS or WBRT. Because these studies influenced committee voting, and are relevant to real-world clinic decision-making, we included data reflecting single-agent activity in Tables 2 and 3.

**Table 2.** Select Single-Agent EGFR TKI Studies Discussed by the Panel

First Author, Design, and Population	BrM Patients (*n*)	BrM Outcomes	LM patients (*n*)	LM Outcomes	Study Quality (SQ)^*^ and Comments
*Gefitinib* (withdrawn from US market in 2011)					
Ceresoli^39^ phase 2 non-selected patients with BrM	41	IC-DCR (PR + SD): 27%	Excluded	NA	SQ: 4 EGFRm not reported 4 systemic therapy naïve pts 44% with prior WBRT
Iuchi^40^ phase 2 EGFRm, TKI naïve, 1–4 BrM	41	IC-ORR: 87.7% mOS in the 15pt with CNS failure: 14.5mo	Excluded	NA	SQ: 3 single arm, allowed switch to erlotinib at progression
*Erlotinib*					
Iuchi^40^ phase 2 EGFRm with CNS progression on gefitinib study above	13	IC-ORR: 58.3% mPFS: 11.9 mo Pts getting gefitinib followed by erlotinib, mOS from BrM diagnosis was 21.9 mo	Excluded	NA	SQ: 3 single arm
Yu^41^ phase 2 EGFRm stage IV or recurrent disease treated with twice weekly pulse + daily dose	A: 11 of the 34 B: 19 treatment-naïve BrM	A: 0/11 with CNS progression on study B: 3/19 with CNS progression on study CNS and systemic ORR: 75 and 74% mPFS: 10 mo	Excluded	NA	SQ: 3 Small subgroup within small single-arm study
*Afatinib*					
Shuler^29^ phase 3 (pooled LUX-LUNG 3 and 6) EGFRm first-line afatinib v. platinum-based chemo	81 of the 709 patients	In 81 BrM pts: mPFS: 8.2 v 5.4 mo, HR 0.50 ORR for afatinib 73% ORR chemo 24%	Excluded	NA	SQ:3 Post hoc analysis Roughly 30% pts w prior WBRT Improved PFS but not OS for BrM pts on afatinib
*Osimertinib*					
Wu^42^ phase 3 (AURA3) osimertinib v. platinum-pemetrexed in patients with progression on 1^st^ generation TKI and T790M EGFRm	144 of the 419 patients (2:1 randomized)	CNS ORR 70% v. 31% CNS mPFS 11.7 v. 5.6 mo, *P* = .004 New CNS lesions: 11 v. 22% CNS Progressions: 16 v. 32% In osi arm, CNS ORR: If CNS RT w/in 6 mo: 64% If Remote or no CNS RT: 34%	7	Pooled analysis by Ahn et al.	SQ: 3 Pre-planned CNS subgroup Blinded central neuroradiology 19% osi and 22% of chemo patients had CNS RT within 6 months prior to randomization
Reungwetwattana^43^ phase 3 (FLAURA) Osimertinib v. gefitinib or erlotinib in newly diagnosed NSCLC (exon19 or L858R EGFRm)	128 of the 556 asymptomatic BrM 41 with measurable lesions	All BrM (measurable and non): ORR 66 v. 43%, OR 2.5, *P* = .011 PFS 15.2 v 9.6 mo HR 0.47, *P* ≤ .001 CNS PFS: NR v 13.9 mo, HR 0.48 In overall study CNS progression events: 6 v. 15%	Allowed (as non-measurable) 7 total 5 osimertinib 2 standard TKI	4/5 in osi arm with CR 1/5 in osi with non-CR/non-PD	SQ: 3 25% of patients in both treatment groups had CNS RT within 6 months prior to randomization Symptomatic BrM allowed if RT then stable for 2 weeks.
Ahn^44^ phase 1 (BLOOM) EGFRm with BrM or LM	29 dose escalation 38 dose expansion	Dose-escalation with target CNS lesions: 21pt; 11 with shrinkage, 3 confirmed, 3 unconfirmed PR Dose-expansion with target CNS lesions: 18 treatment-naïve pts; 14 PRs, 1 CR	22 in dose expansion	TKI Naïve (4): 2 CR, 1 PR Prior TKI (18): 1 CR, 4PR, 9 SD	SQ:3 Multiple cohorts Other outcomes reported later Unclear outcome measure for LM
Ahn^45^ retrospective pooled analysis of patients receiving Osimertinib for LM on AURA series studies	N/A- LM only	NA	22	ORR 55% by RANO-LM mPFS 11.1mo	SQ: 3 Includes AURA extension, AURA2, AURA3, AURA17
Yamaguchi^33^ phase 2 (OCEAN) EGFRm T790M new BrM	39 RT Naïve	CNS-ORR 66.7% by PAREXEL CNS-ORR 70% by RECIST CNS-PFS at 25.2 mo	Not specifically reported	NA	SQ:3 Small, non-randomized study without RT comparator arm
Janne^46^ phase 3 (FLAURA2) Osimertinib v. Osimertinib with platinum-pemetrexed first line	224 of the 568 78 with measurable lesions	All CNS mets CNS-PFS 20.1 mo (combo) v 13.9 mo CNS-ORR 73% (combo) v 69% Measurable disease CNS-PFS 30.2 mo (combo) v 27.6 CNS-ORR 88% (combo) v 87%	13 combo 5 osimertinib alone	9 in combo arm with response (5 CR) 2 in mono with response (1 CR)	SQ: 3 Subset of large, randomized study. Approx 15% with prior RT LM response criteria not clear
*Lazertinib*					
Passaro^47^ phase 3 (MARIPOSA-2) Amivatinib + chemo with or without Lazertinib v chemo Progression on osimertinib	298 of the 657	CNS-PFS chemo 8.3 mo CNS-PFS ami-chemo 12.5 mo CNS-PFS ami-chemo-lazer 12.8 mo	Not explicitly excluded	Not reported	SQ: 3 Approx 55% with prior RT Ami and ami + lazer arms with high toxicity Supports ami as possible CNS-active agent

^*^Study quality ratings are based on how this study relates to the KQs and are not intended to comment on the overall strength of the study.

Study quality category definitions.

*Category 1* The study is well-designed and accounts for common biases.

*Category 2* The study is moderately well-designed and accounts for the most common biases.

*Category 3* There are important study design limitations.

*Category 4* The study is not useful as primary evidence. The article may not be a clinical study, or the study design is invalid, or conclusions are based on expert consensus. For example:

(a) the study does not meet the criteria for or is not a hypothesis-based clinical study (eg, a book chapter or case report or case series description).

(b) the study may synthesize and draw conclusions about several studies such as a literature review article or book chapter but is not primary evidence.

(c) the study is an expert opinion or consensus document.

*Category M* The study is a meta-analysis and has not been rated for quality because the method is designed to evaluate individual studies only.

**Table 3.** Select Single-Agent ALK TKI Studies Discussed by the Panel

First Author, Design, & Population	BrM Patients (*n*)	BrM Outcomes	LM patients (*n*)	LM Outcomes	Study Quality (SQ)^*^ and Comments
*Ceretinib*					
Kim^48^ phase 1 (ASCEND-1) ALK TKI naïve and pretreated	94	ALK naïve IC-DCR 78.9% ALK pretreated IC-DCR 65.3% PR in 6/11 (55%) measurable & no prior RT	1	Not reported	SQ:3 CNS outcomes were retrospectively reviewed and reported in 2016
Crino^49^ phase 2 (ASCEND-2) progressive disease on crizotinib	100	20 with target lesion IC-ORR 45% IC-DCR 80%	Excluded	NA	SQ: 3 Target lesions were either treatment-naïve or considered progressed after RT, though status not reported
Nishio^50^ phase 2 (ASCEND-3) ALK TKI naïve	49	22% had previous brain RT All BrM PFS: 10.8 mo (95% CI: 7.3–16.6) In 13 patients with measurable disease IC-ORR 61.5% IC-DCR 76.9%	Excluded	NA	SQ:3 Target lesions were either treatment naïve or considered progressed after RT
Soria^51^ phase 3 (ASCEND-4) first-line ALK TKI v. platinum-pemetrexed	121	44 pts (22 in each group) with active BrM and post-baseline assessment PFS for all pts with BrM by independent review 10.7 v 6.7 mo (HR 0.7, [95% CI: 0·44–1·12]) IC-ORR in active, measurable BrM 72.7 v 27.3%	Excluded	NA	SQ:3 Approximately 90% of previously treated BrM had RT in ≤3 months from randomization
Shaw^52^ phase 3 (ASCEND-5) Ceretinib v. chemotherapy in ALK disease progressed on chemotherapy and crizotinib	133	57% of pts with prior brain RT 37 patients with active, measurable lesions and follow-up IC-ORR 35% v 5% IC-DOR 6.9 mo in ceritinib (not accessible in chemo arm)	Excluded	NA	SQ:3
Wu^53^ phase 1/2 (ASCEND-6) previously treated patients in China	65	23 with measurable disease 7 with prior RT In measurable: IC-ORR 39.1% IC-DCR 82.6%	Excluded	NA	SQ:3
Chow^54^ phase 2 (ASCEND-7) ALK with metastases to brain or leptomeninges	138	44 pt in Arm 4: no prior RT, ALK naive In 33 patients with measurable disease: IC-ORR: 51.5 [33.5, 69.2] IC-DCR: 75.8 [57.7, 88.9] DOR (17 pts): 7.5 mo [5.6, 11.2]	Arm 5: 18	IC-ORR: 12.5 (0.3–52.7) IC-DCR: 62.5 (24.5–91.5)	SQ:3 1 patient with DOR in Arm 5 Primary endpoint was whole-body ORR
*Alectinib*					
Gadgeel^55^ phase 1 Resistant or intolerant to crizotinib	21 of the 44	All BrM pts: IC-ORR 52% 12 with active BrM 4 patients with no prior RT: 2CRs, 1 PR, 1SD 1/44 had CNS progression at 5-month follow-up	1 (symptomatic LM excluded)	“favorable activity”	SQ:3 Median time from prior brain RT was 4 months
Shaw^56^ phase 2 ALK with progression on crizotinib	52/87 (60%)	In all BrM: IC-ORR 40% IC-DOR 11.1 months [95% CI 10·8–not estimable] In 18 (35%) without prior RT, IC-ORR 67%.	0, symptomatic LM excluded	NA	SQ:3
Hida^57^ phase 3 (J-ALEX) alectinib vs. crizotinib in ALK naïve patients who had or had not received one prior chemotherapy	43/207 (21%)	Crizotinib: 29pt (28%) Alectinib: 14pt (14%) HR for time to CNS progression/death in BrM patients: 0·16 (0·02–1·28) HR for time to CNS progression/death in non-BrM patients: 0·41 (0·17–1·01)	0, symptomatic LM excluded	NA	SQ:3 Imbalanced for BrM (not stratified) Not all patients had baseline brain imaging
Peters^58^ phase 3 (ALEX) alectinib vs. crizotinib in previously untreated advanced ALK disease	122/303 (42%)	Crizotinib: 58 (38%) Alectinib: 64 (42%) Measurable disease: IC-ORR 81% v 50% IC-DOR 17.3 mo v 5.5 12-mo cumulative incidence of CNS progression 9.4% v 41.4%	Not reported, symptomatic LM excluded	NA	SQ:3 Approx 15% of patients with prior brain RT Data from 2017 cutoff (there is a 2020 update)
*Brigatinib*					
Gettinger^59^ phase 1/2 advanced refractory ALK	50/79 (63%) NSCLC-ALK Cohort 5 (active, measurable):6 pts	23/50 (46%) no prior RT and of 21 assessable IC-ORR 57% (measurable + non-measurable) and IC-mPFS: 23 mo Cohort 5: IC-ORR 50%	Not reported, Symptomatic neurodisease excluded	NA	SQ: 3 Full study includes other disease types
Huber^60^ phase 2 (ALTA) progressive ALK disease on ceritinib or alectinib	Arm A (90 mg): 80/112 (71%) Arm B (180 mg): 74/110 (67%)	Arm A: Active lesions 54/112 (48%) IC-ORR measurable 13/26 (50%) IC-DOR 9.4 m Arm B: Active lesions 55/110(50%) IC-ORR measurable 12/18 (67%) IC-DOR 16.6 mo	Not reported, Symptomatic neurodisease excluded	NA	SQ:3 61% Arm A and B with prior brain RT
Camidge^61^ phase 3 (ALTA-1L) Brigantinib vs. crizotinib ALK naïve advanced disease	90 (33%) overall	Brigatinib 40 (29%) Crizotinib 41 (30%) In BrMs, CNS-PFS-12 mo: 67% brigantinib 21% crizotinib In 39 with measurable disease: IC-ORR 78% v 29%	Not reported, Symptomatic neurodisease excluded	NA	SQ:3 Approximately 26% in both groups with prior brain RT
*Lorlatinib*					
Solomon^62^ phase 1/2 advanced, progressive ALK disease	EXP1: treatment-naive 8/30 (27%) EXP2-5: ≥ 1 prior ALKi w or wo chemo 133/198 (67%)	Activity in measurable disease: EXP1 IC-ORR 67% (2/3) IC-DOR NR EXP2-5 IC-ORR% 63% (51/81) IC-DOR 14.5 mo	Asymptomatic LM allowed but none explicitly reported	NA	SQ:3 EXP1: 25% (2/8) prior brain RT EXP2-5: 65% (86/133) prior brain RT
Shaw^63^ phase 3 (CROWN) first-Line lorlatinib vs. crizotinib	lorlatinib 38/149 (26%) crizotinib 40/147 (27%)	In measurable disease: IC-ORR 66% v 20% IC-DOR NR v 10.2 mo	Asymptomatic LM allowed but none explicitly reported	NA	SQ:3 Prior brain RT in about 7% of both groups
Dagogo-Jack^64^ phase 2 isolated CNS relapse on prior ALKi	23	IC-ORR 59% IC-PFS 24.6 mo	4 with LM and BrM	“best response was stable disease”	SQ:3 New or progressing BrM required
*Evonalkib*					
Yang ^65^ phase 3 first line evonalkib vs. crizotinib	88 of the264 40 measurable	In target lesions IC-ORR 78.95% v 23.81% CNS-TTP 26.68 mo v 6.34 mo Incidence of new BrM 2.15 v 11.7%	Not reported	NA	SQ:3 Asymptomatic BrM only; does not report if BrMs had prior w RT

^*^Study quality ratings are based on how this study relates to the KQs and are not intended to comment on the overall strength of the study.

Study Quality Category Definitions.

*Category 1* The study is well-designed and accounts for common biases.

*Category 2* The study is moderately well-designed and accounts for most common biases.

*Category 3* There are important study design limitations.

*Category 4* The study is not useful as primary evidence. The article may not be a clinical study, or the study design is invalid, or conclusions are based on expert consensus. For example:

(a) the study does not meet the criteria for or is not a hypothesis-based clinical study (eg, a book chapter or case report or case series description).

(b) the study may synthesize and draw conclusions about several studies such as a literature review article or book chapter but is not primary evidence.

(c) the study is an expert opinion or consensus document.

*Category M* The study is a meta-analysis and has not been rated for quality because the method is designed to evaluate individual studies only.

Table 2 reviews selected EGFR studies with a focus on CNS outcomes. Most studies were limited by design, small patient numbers, and incomplete data about which patients and BrM received prior radiotherapy. Studies of first-generation drugs erlotinib and gefitinib were also limited by lack of testing for EGFRm. The second-generation EGFR TKIs, afatinib, and dacomitinib, are irreversible blockers of EGFR. Both drugs demonstrated increased efficacy compared to first-generation EGFR TKIs. However, most dacomitinib studies, including the pivotal phase 3 (ARCHER 1050), excluded patients with BrM.^66^ The early development of afatinib either excluded patients with BrM or did not explicitly report outcomes for those with asymptomatic BrM. A later pooled analysis of 81 patients enrolled on LUX-Lung 3 and 6, and who were asymptomatic from BrM, demonstrated a PFS and overall response rate (ORR) benefit for patients receiving afatinib, but no benefit in mOS.^29^

Development of third-generation EGFR TKIs osimertinib and rociletinib included substantially more BrM patients than their predecessors. These drugs were designed to overcome resistance mechanisms to first- and second-generation drugs. Though rociletinib was not FDA approved, of 160 patients in the phase 1–2 program and 149 on the incomplete TIGER-3 phase 3, approximately 42% had (treated, asymptomatic, stable) BrM or a history of BrM at enrollment.^67,68^ Data from the phases 1 and 2 studies of osimertinib also supported the inclusion of patients with asymptomatic BrM into the phase 3 AURA and FLAURA studies. While FLAURA and AURA compared osimertinib to other systemic therapies, and did not directly compare osimertinib to radiotherapy, patients receiving osimertinib had improved CNS ORR, PFS, OS, and a reduced number of CNS progression events.^32,69^ A smaller, non-randomized study (BLOOM) of osimertinib in patients with CNS metastases also demonstrated clear activity in patients with LM.^35^ Data from the T790M cohort of the OCEAN study became available during later voting rounds and was discussed by the committee because it specifically evaluated osimertinib in patients with radiation-naive BrM. In 39 patients with a T790M EGFRm and new BrM, using the novel PAREXEL BrM criteria, the IC-RR was 66.7%; a subset had RECIST measurable disease and had an IC-RR of 70%. While in FLAURA, the CNS PFS had not been reached with median of 12.4 months of follow-up, the OCEAN study reported a 25.2-month BrM-related PFS.^33^ These data informed the committee’s recommendation that the use of a third-generation CNS penetrant TKI in lieu of radiotherapy *may be appropriate*. In December 2023, the CNS data from FLAURA2, a randomized phase 3 trial of first-line osimertinib plus chemotherapy versus osimertinib alone, were published. While the CNS ORR was similar in both groups (≅70%), patients in the combination group experienced more CNS-CRs and longer DOR (93% vs. 81% alive and in CNS response at 12 months, combination vs. monotherapy). As expected, the incidence of grade ≥3 adverse events was higher in the combination group. How the balance of improved PFS against increased side effects alters the choice of first-line therapy for patients with metastatic EGFRm NSCLC is not yet clear. Because it does not directly address the role of radiation, the committee felt it would not alter voting on the key question of deferring radiation in lieu of systemic therapy. Similarly, data from FURLONG (furmonertinib versus gefitinib)^70^ was reviewed, but not considered applicable to the KQs.

Table 3 reviews selected studies of ALK inhibitors with a focus on CNS outcomes. The first commercially available ALK inhibitor, crizotinib, received accelerated approval by the FDA in 2011 after 2 small multicenter trials that demonstrated high ORRs (50%–60%) and response durations of 42–48 weeks. However, a higher-than-expected proportion of patients developed CNS progression; in 10 of the 39 patients on extended treatment, the only site of disease progression was brain.^71^ Despite this, the phase 3 PROFILE 1014 study of crizotinib versus platinum-pemetrexed included patients with stable BrM and a preplanned CNS efficacy endpoint, time to intracranial progression (IC-TTP). Though there were no differences in IC-TTP, patients who had previously treated BrM receiving crizotinib had significantly longer intracranial-disease control rate (IC-DCR) than patients on the platinum-pemetrexed arm (56% v. 25% at 24 weeks, *P* = .006).^72^

The development programs for second-generation drugs alectinib, ceritinib, and brigatinib included specific BrM cohorts, though the number of patients with previously untreated, measurable disease remained small. The pivotal phase 3 study of alectinib (ALEX) included 122 patients with BrM and demonstrated a substantially lower 12-month cumulative incidence rate of CNS progression with alectinib arm versus crizotinib (9.4% vs. 41.4%).^58^ In the 2-arm, open-label ALTA study of 90 or 180 mg of brigantinib daily, approximately 70% of 220 patients had baseline BrM. Roughly half had radiation naïve BrM or progressive disease after radiotherapy. Both arms demonstrated intracranial activity, but the 180 mg dose level had an independent radiology-confirmed IC-ORR of 67% in patients with measurable lesions, a median IC-DOR of 16.6 months, and median IC-PFS of 18.4 months, the longest reported responses of any CNS-active TKI.^60^ Lorlatinib is a third-generation ALK inhibitor, and the most recently approved. Patients with BrM and LM were allowed to enroll in all phases of the study and IC-RRs across these studies were between 60% and 70% with IC-DORs > 14 months.^62,63^ Data from BrM patients in the phase 3 study of envonalkib versus crizotinib fall in a similar range.^65^ The data for patients with BrM and LM support the ARS panel’s recommendation that the use of ALK TKIs alone in lieu of radiotherapy *may be appropriate*.

*KQ2: If radiation therapy is recommended (SRS or WBRT), should it be given together with TKI or in sequence?*

Within the 8 cases, 22 scenarios directly addressed KQ2. WBRT with concurrent TKI was considered *usually not appropriate* in 3 of the 22 scenarios, based on the *in*appropriateness of WBRT for 2–4 BrMs, rather than the use of TKI. In the remaining cases, the use of WBRT with a concurrent TKI generated broad disagreement, defaulting to a *may be appropriate* recommendation (Table 4).

**Table 4.** Panelist Voting on Treatment Scenarios Addressing KQ2: the Use of TKI Concurrently, With Radiation. All Cases Included WBRT. Some Included Special Circumstances, Such as SRS Boost (Included in Italics)

Clinical Scenario (Case variant)	Agreement or Disagreement	Vote Breakdown	Recommendation	SOR	SOE
Newly diagnosed EGFR/ALK NSCLC with 2–4 asymptomatic BrM (CV 1a)	Agreement with recommendation based on median rating (1.5)	2 votes outside of usually not appropriate category	*Usually not appropriate*	Strong	Strong^27^
Newly diagnosed EGFR/ALK NSCLC with 5 to >20 asymptomatic BrM (CV 1b,c,d)	Disagreement	Broadly distributed across 3 categories	*May be appropriate*	Conditional	Expert opinion^*^
EGFR/ALK NSCLC with systemic progression on first/second generation TKI, 2–4 asymptomatic new BrM (CV 2a)	Agreement with recommendation based on median rating (2)	2 votes outside of usually not appropriate category	*Usually not appropriate*	Strong	Strong^27^
EGFR/ALK NSCLC with systemic progression on first/second generation TKI, 5–10 asymptomatic new BrM (CV 2b)	Disagreement	Broadly distributed across 3 categories	*May be appropriate*	Conditional	Expert opinion^*^
EGFR/ALK NSCLC with systemic progression on first/second generation TKI, 11–20 asymptomatic new BrM (CV 2c)	Agreement with recommendation based on median rating (4)	Distributed, but most voters in may be appropriate	*May be appropriate*	Conditional	Expert opinion^*^
EGFR/ALK NSCLC with systemic progression on first/second generation TKI, >20 asymptomatic new BrM (CV 2d)	Disagreement	All votes in maybe or usually appropriate	*May be appropriate*	Conditional	Expert opinion^*^
Newly diagnosed EGFR/ALK NSCLC with 2 symptomatic, unresectable brain metastases (C3)	Agreement with recommendation based on median rating (3)	Majority is usually not appropriate	*Usually not appropriate*	Strong	Strong^27^
Newly diagnosed EGFR/ALK NSCLC with 2 symptomatic, unresectable brain metastases (C3) *with SRS boost*	Disagreement	Evenly split is usually not appropriate and may be appropriate	*May be appropriate*	Weak	Expert opinion^*^
Newly diagnosed EGFR/ALK NSCLC with multiple brain metastases, 2 of larger size and symptoms at the time of diagnosis (C4)	Disagreement	Broadly distributed across 3 categories	*May be appropriate*	Conditional	Expert opinion^*^
Newly diagnosed EGFR/ALK NSCLC with multiple brain metastases, 2 of larger size and symptoms at time of diagnosis (C4) *with SRS boost*	Disagreement	Broadly distributed across 3 categories	*May be appropriate*	Conditional	Expert opinion^*^
Isolated CNS progression in EGFR/ALK NSCLC while on third-generation CNS penetrant drug, unknown CNS mutation profile (CV 5a)	Agreement with recommendation based on median rating (4)	Most votes in may be appropriate	*May be appropriate*	Conditional	Expert opinion^*^
Isolated CNS progression in EGFR/ALK NSCLC while on third-generation CNS penetrant drug, new CNS mutation is targetable w TKI (CV 5b) *standard or high dose TKI to follow*	Disagreement	Broadly distributed across 3 categories	*May be appropriate*	Conditional	Expert opinion^*^
Isolated CNS progression in EGFR/ALK NSCLC while on third-generation CNS penetrant drug, new CNS mutation is non-targetable (CV 5c) *standard or high dose TKI to follow*	Disagreement	Broadly distributed across 3 categories	*May be appropriate*	Conditional	Expert opinion^*^
Isolated CNS progression in EGFR/ALK NSCLC while on third-generation CNS penetrant drug, CNS mutation profile is unchanged from original (CV 5 d)	Disagreement	Distributed across 3 categories, but clustered around may be appropriate	*May be appropriate*	Conditional	Expert opinion^*^
EGFR/ALK NSCLC with modestly symptomatic diffuse LM that developed on first or second gen TKI (CV 7b)	Disagreement	Distributed across 3 categories, but clustered around may be appropriate	*May be appropriate*	Conditional	Expert opinion^*^
Newly diagnosed NSCLC with targetable mutation (EGFR or ALK) with symptomatic diffuse LM (CV 7c)	Disagreement	Distributed across 3 categories	*May be appropriate*	Conditional	Expert opinion^*^
EGFR/ALK NSCLC with significantly symptomatic diffuse LM + BrM that developed on third-generation TKI (CV 7d)	Disagreement	Distributed across 3 categories	*May be appropriate*	Conditional	Expert opinion^*^
Progressive EGFR/ALK NSCLC on third-generation TKI with 12 asymptomatic sub-cm BrM *New TKI CNS RR < 50%, mDOR 6 mo* (CV 8a)	Agreement with recommendation based on median rating (5)	Distributed across 3 categories	*May be appropriate*	Conditional	Expert opinion^*^
Progressive EGFR/ALK NSCLC on third-generation TKI with 12 asymptomatic sub-cm BrM *All 4 scenarios where new TKI CNS RR > 50%, and mDOR ≥6 mo* (CV 8b-e)	Disagreement	Distributed across 3 categories	*May be appropriate*	Conditional	Expert opinion^*^

^*^The evidence for this recommendation is based on extrapolations from retrospective data and meta-analysis. These treatment scenarios are not directly covered in an individual manner by specific studies.

### Evidence to Support Decision-Making for KQ2

Twelve studies from the evidence table addressed KQ2, but none were randomized, prospective trials directly addressing the question of concurrent versus adjuvant TKI in patients with CNS disease. A subgroup analysis of EGFRm patients in phase 3 randomized study of WBRT with or without erlotinib in non-selected NSCLC patients was published in 2021. The panel reviewed the data and felt this supported safety of the combination but not increased efficacy.

In 2004, positive results from a phase 3 trial of EGFR-targeted antibody, cetuximab with concurrent radiotherapy for head and neck cancer suggested EGFR inhibitors may be radiosensitizers.^73^ The earliest EGFR-TKI/WBRT combination studies were occurring simultaneously. Unfortunately, EGFRm was either not tested or not reported for most patients.^74–77^ Even the RTOG study that included a WBRT plus erlotinib arm was done prior to selection of EGFRm patients.^78^ Therefore, though these studies confirmed safety of the combinations, it’s difficult to draw conclusions about efficacy in the EGFRm population. Later studies included small groups of patients who had EGFR testing. In Zhuang et al’s phase 2 study of WBRT with or without erlotinib, only patients with EGFR testing (not necessarily mutations) received erlotinib.^79^ Though the combination arm had better IC-PFS, PFS, and OS than patients who received WBRT alone, there were no significant endpoint differences between EGFRm and EGFRwt patients. The follow-up phase 3 trial used the same non-selective enrollment criteria. The EGFRm subgroup analysis (109 patients) demonstrated no significant difference in IC-PFS in patients randomized to concurrent versus sequential WBRT and TKI.^80^ Our literature search did not reveal any prospective studies of ALK inhibitors and WBRT. Published data demonstrate the safety of concurrent TKI and WBRT, but do not demonstrate a consistent advantage in patient outcomes.

*KQ3: At the time of CNS progression on TKI, should RT be deferred if another CNS-penetrating TKI is available?*

KQ3 was addressed in 15 management scenarios (Table 5). Using currently available TKIs, the panel did not find consensus in any scenario involving CNS progression on a previous TKI that deferring radiotherapy for BrM could be considered *usually appropriate*. The committee was more likely to agree that deferral of radiation *may be appropriate* if a higher dose of TKI could be given or the TKI could be combined with an agent targeting a resistance mutation. Only in the case of a theoretical new TKI with CNS RR of ≥80% and DOR ≥14 months did the panel find the deferral of RT in favor of TKI *usually appropriate*. In the setting of diffuse and asymptomatic LM (CV7a) occurring as recurrence, TKI alone was thought to be *usually appropriate* by most panelists.

**Table 5.** Panelist Voting on Treatment Scenarios Addressing KQ3: Use of TKI Alone, in Lieu of Radiation, in Recurrent NSCLC. In These Scenarios, TKI Dose and Combinations Were Explored

Clinical Scenario (Case or Case Variant)	Treatment	Agreement or Disagreement	Vote Breakdown	Recommendation	SOR	SOE
EGFR/ALK NSCLC with systemic progression on first/second generation TKI, 2–10 asymptomatic new BrM (CV 2a and b)	Third-generation TKI alone	Disagreement	One vote not appropriate, all others may be or are usually appropriate	*May be appropriate*	Conditional	Moderate^43,44,45,58,59^
EGFR/ALK NSCLC with systemic progression on first/second generation TKI, 11 to >20 asymptomatic new BrM (CV 2c and d)	Third-generation TKI alone	Disagreement	Distributed across may be and usually appropriate	*May be appropriate*	Conditional	Expert opinion^*^
Isolated CNS progression in EGFR/ALK NSCLC while on third-generation CNS penetrant drug, unknown CNS mutation profile (CV 5a)	Third-generation TKI alone at higher dose	Agreement, with recommendation based on median rating (5)	Distributed across all 3 categories, with most in may be appropriate	*May be appropriate*	Conditional	Expert opinion^*^
Isolated CNS progression in EGFR/ALK NSCLC while on third-generation CNS penetrant drug, new CNS mutation is targetable w TKI (CV 5b)	Third-generation TKI alone at higher dose	Agreement, with recommendation based on median rating (5)	Distributed across may be appropriate and not usually appropriate	*May be appropriate*	Conditional	Expert opinion^*^
Isolated CNS progression in EGFR/ALK NSCLC while on third-generation CNS penetrant drug, new CNS mutation is targetable w TKI (CV 5b)	Switch to new TKI alone	Disagreement	Distributed across may be appropriate and usually appropriate	*May be appropriate*	Conditional	Expert opinion^*^
Isolated CNS progression in EGFR/ALK NSCLC while on third-generation CNS penetrant drug, new CNS mutation is targetable w TKI (CV 5b)	Combine third-generation and new TKI	Agreement, with recommendation based on median rating (5)	Votes are clustered in may be appropriate	*May be appropriate*	Conditional	Expert opinion^*^
Isolated CNS progression in EGFR/ALK NSCLC while on third-generation CNS penetrant drug, new CNS mutation is non-targetable (CV 5c)	Continue third-generation TKI alone, at higher dose	Agreement, with recommendation based on median rating (4)	All votes in may be appropriate and not usually appropriate	*May be appropriate*	Conditional	Expert opinion^*^
Isolated CNS progression in EGFR/ALK NSCLC while on third-generation CNS penetrant drug, CNS mutation profile is unchanged from original (CV 5d)	Continue third-generation TKI alone, at higher dose	Agreement, with recommendation based on median rating(6)	Single vote in not usually appropriate	*May be appropriate*	Conditional	Expert opinion^*^
EGFR/ALK NSCLC with modestly symptomatic diffuse LM that developed on first or second-generation TKI (CV 7b)	Third gen TKI alone	Agreement, with recommendation based on median rating (5.5)	All votes within may be and usually appropriate	*May be appropriate*	Conditional	Limited^34,81^
EGFR/ALK NSCLC with significantly symptomatic diffuse LM + BrM that developed on third-generation TKI (CV 7d)	Continue third gen TKI alone, at higher dose	Agreement, with recommendation based on median rating (5)	Single vote in not usually appropriate	*May be appropriate*	Conditional	Expert opinion^*^
Progressive EGFR/ALK NSCLC on third-generation TKI with 12 asymptomatic sub-cm BrM (CV 8a)	New TKI CNS RR < 50%, mDOR 6 mo	Agreement, with recommendation based on median rating (5)	Distributed across all 3 categories, with most in may be appropriate	*May be appropriate*	Conditional	Expert opinion^*^
Progressive EGFR/ALK NSCLC on third-generation TKI with 12 asymptomatic sub-cm BrM (CV 8b)	New TKI CNS RR 50%, mDOR 8 mo	Agreement, with recommendation based on median rating (5)	Distributed across all 3 categories, with most in may be appropriate	*May be appropriate*	Conditional	Expert opinion^*^
Progressive EGFR/ALK NSCLC on third-gen erationTKI with 12 asymptomatic sub-cm BrM (CV 8c)	New TKI CNS RR 65%, mDOR 8 mo	Disagreement	Distributed across all 3 categories, with most in may be appropriate or usually appropriate	*May be appropriate*	Conditional	Expert opinion^*^
Progressive EGFR/ALK NSCLC on third-gen erationTKI with 12 asymptomatic sub-cm BrM (CV 8d)	New TKI CNS RR 80%, mDOR 14 mo	Agreement, with recommendation based on median rating (7)	All votes within may be and usually appropriate	*Usually appropriate*	Conditional	Expert opinion^*^
Progressive EGFR/ALK NSCLC on third-gen erationTKI with 12 asymptomatic sub-cm BrM (CV 8e)	New TKI CNS RR 95%, mDOR 18 mo	Agreement, with recommendation based on median rating (8)	Single vote in may be appropriate, remainder usually appropriate	*Usually appropriate*	Conditional	Expert opinion^*^

^*^The evidence for this recommendation is based on extrapolations from retrospective data and meta-analysis. These treatment scenarios are not directly covered in an individual manner by specific studies.

### Evidence to Support Decision-Making for KQ3

Four papers from the evidence table addressed KQ3. There were no randomized phase 3 studies comparing radiotherapy to switching TKIs at the time of intracranial progression. A prospective, randomized phase 2 study compared 2 doses of brigatinib in a population that included patients with BrM and demonstrated single-agent activity in patients whose disease had progressed on crizotinib, but was not randomized against radiotherapy.^60^ Data from MARIPOSA-2 (amivantamab with chemotherapy with or without lazertinib) support the use of the combination after osimertinib, including in patients with BrM, but did not solidify lazertinib over amivantamab as the most active CNS agent, nor did it compare the regimens to salvage RT.^82^ Because studies of TKI versus RT for CNS progression were not identified, the committee referred to the data available in Tables 2 and 3 to support decision-making.

### Special Case Variants

Within the 8 cases developed by the panel, management options including pre-operative SRS, craniospinal RT (CSI), and hospice/best supportive care were addressed. Two cases allowed a pre-operative SRS option (C4 & C6). In both scenarios, all votes fell within *may be* or *usually appropriate* categories, with discussion centered around possible reduction of LM after SRS. NRG BN012 is a randomized trial of pre- or post-operative SRS for BrM, designed to determine if pre-operative SRS reduces the incidence of LM. Results from this study may lead to consensus recommendations surrounding the timing of surgery and SRS.

Case 7 included 4 variations of symptomatic and asymptomatic LM. For patients with no or minimal symptoms, the committee agreed that TKI alone or combined with selective SRS *may be appropriate*. The BLOOM study of patients with LM from EGFRm NSCLC as well as exploratory cohorts of patients with LM in larger studies of ALK + NSCLC supported this decision-making. Treatment options for these cases also included CSI or hospice/best supportive care. Surprisingly, there was disagreement about hospice/best supportive care across all 4 variants, though the majority of votes fell within *may be appropriate* for hospice in all scenarios. There was also widespread disagreement about the use of photon CSI, owing to a lack of compelling data and shared clinical experience. Notably, the committee debated the addition of proton CSI as a treatment option, but opted against it as access to protons remains limited and the study from Yang et al. was not available at the time of case formulation. This randomized phase 2 study of patients with LM from breast or lung cancer found proton CSI improved CNS PFS (primary endpoint) and OS compared to photon WBRT with select, focal spine RT.^30^ Only 21 (21%) of patients in the study had EGFR or ALK NCLSC, they were not evenly distributed, and the study did not randomize to TKI alone or photon CSI. Panel discussion indicates this study may have raised support for CSI overall, but it does not address the question of choosing TKI alone over any form of CSI.

In case 8, we asked members to assume access to a hypothetical new TKI with varying CNS response rates and DOR for an asymptomatic patient with 12 sub-centimeter BrM. With increasing CNS response rate and DOR, votes for using TKI alone increasingly clustered in the *usually appropriate* category with clear agreement that TKI alone would be appropriate in most cases if the drug had an ≥80% CNS response rate and DOR ≥14 months. At and above this inflection point, there was disagreement about the use of SRS with TKI, whereas with lower CNS RR and DOR, there was agreement that SRS followed by TKI was *usually appropriate*. This is consistent with current performance expectations for SRS, and thereby for a therapy warranting deferral of radiation.^31,34^ The ARS-AUC committee hopes that these cases demonstrate a patterned approach to the use of new therapies with CNS activity.

## Discussion

The management of BrM and LM in patients with NSCLC containing targetable alterations is evolving rapidly as the number of CNS penetrant systemic treatments increases. However, a comprehensive review of the literature published through December 2023 did not reveal any study that provided definitive recommendations for the optimal timing of radiotherapy and TKI in the treatment of BrM or LM from EGFRm or ALK + NSCLC. The only randomized study between radiotherapy and TKI compared WBRT with a first-generation drug that does not have US-FDA approval and did not demonstrate a survival difference. The committee reviewed multiple prospective studies of patients with BrMs receiving EGFR and ALK TKIs that confirmed CNS activity in the absence of radiotherapy but did not compare TKI alone to radiotherapy directly. These studies included a mix of patients with radiation naïve and pretreated BrM, but outcomes were rarely reported separately. Some studies included only measurable disease by RANO or RECIST, requiring a ≥1 cm target, which the committee agreed, does not reflect current clinical practice, where smaller metastases are commonly treated. Differing outcome measures also make cross-study comparisons challenging. For example, drug studies tend to report response rates (often as a percent of total diameters) and DOR on an overall CNS level, whereas radiation oncology studies report local versus distant/elsewhere CNS control rates. As a result, how studies were interpreted varied across committee members leading to a frustrating situation, with the committee unable to come to consensus or agreement in many treatment scenarios, even after multiple rounds of discussion and voting. Interestingly, a multidisciplinary panel from Spain (5 medical oncologists, 2 radiation oncologists, and 1 neuro-oncologist) used a similar guideline methodology based on literature up to 2019 but reached 100% agreement/consensus that it is reasonable to postpone or delay the use of radiation in patients with asymptomatic and minimally symptomatic BrM.^81^ The make-up of our committee, more heavily weighted to radiation oncology, may account for some of the difference. Additionally, access to SRS in Europe is often more limited than in the United States. Data collected from Spain after adoption of these guidelines may further influence future ARS guidelines.

The important clinical questions raised here could be answered by well-designed phase 3 randomized studies comparing radiotherapy to a systemic agent alone in patients with treatment-naïve CNS disease. Such studies should clearly define active or pretreated brain lesions, consider that a 1 cm metastases may be prohibitive for enrollment, and use response criteria that are meaningful both for radiation oncologists and medical/neuro-oncologists alike. Ideally, the reporting of neurologic OS should also be considered. To this end, the committee eagerly anticipates the results of NCTs 03769103 and 03497767; both studies plan to randomize patients with EGFRm NSCLC and BrM to osimertinib alone versus osimertinib + SRS. Outcomes will include time to SRS or WBRT and both neurocognitive and quality of life measures. A similar study in ALK rearrangement patients is being planned by the Hoosier Cancer Research Network (NCT05987644).

In the absence of definitive studies, the inclusion of BrM cohorts early in drug development, with attention to prespecified CNS outcome measures will be important in guiding decision-making for patients with BrM. The panel recognizes that the challenges in BrM clinical trials are magnified in LM, but advocates for their continued inclusion in small, exploratory cohorts. LM cohorts also offer a unique opportunity to define CSF and CNS pharmacokinetics and dynamics.

## Summary and Conclusion

The ARS-AUC committee sought to formulate practical guidelines for the treatment of patients with BrM from EGFRm and ALK + NSCLC. After a thorough literature review and case-based discussion, the panel did not reach consensus that CNS radiotherapy can be deferred in patients who can receive a CNS penetrant TKI. Median vote scores, however, indicated the panel felt deferral of radiotherapy in a patient who can receive a CNS penetrant TKI *may be appropriate* under many circumstances. The inability of the ARS-AUC committee to reach consensus reflects the paucity of well-designed, randomized prospective studies that directly address the timing of radiotherapy and TKI. Until these studies are done the committee hopes these cases prompt multidisciplinary discussion and decision making. Ideally, these patients should remain under close clinical and imaging surveillance and have easy access to a team that includes a medical oncologist, neuro-oncologist, radiation oncologist, and neurosurgeon, to address progressive disease.

## Supplementary material

Supplementary material is available online at *Neuro-Oncology* (https://academic.oup.com/neuro-oncology).

noae041_suppl_Supplementary_Data_S1

noae041_suppl_Supplementary_Data_S2
