# Corrigendum to: Executive summary of the American Radium Society appropriate use criteria for brain metastases in epidermal growth factor receptor mutated-mutated and ALK-fusion non-small cell lung cancer

**DOI:** 10.1093/neuonc/noae197

**Published:** 2024-09-21

This is a corrigendum to: Seema Nagpal, Michael T Milano, Veronica L Chiang, Scott G Soltys, Alexandria Brackett, Lia M Halasz, Amit K Garg, Arjun Sahgal, Manmeet S Ahluwalia, Martin C Tom, Joshua D Palmer, Jonathan P S Knisely, Samuel T Chao, Melanie Hayden Gephart, Tony J C Wang, Simon S Lo, Eric L Chang, Executive summary of the American Radium Society appropriate use criteria for brain metastases in epidermal growth factor receptor mutated-mutated and ALK-fusion non-small cell lung cancer, *Neuro-Oncology*, Volume 26, Issue 7, July 2024, Pages 1195–1212, https://doi.org/10.1093/neuonc/noae041

In the originally published version of the manuscript there was an error in the 12th author’s last name which is now corrected to read: “Jonathan P S Knisely”.

The emendation has been made to the article.

