# Supplementary material for: Executive summary of the American Radium Society appropriate use criteria for brain metastases in epidermal growth factor receptor mutated-mutated and ALK-fusion non-small cell lung cancer
Source: Neuro Oncol. 2024 Mar 9;26(7):1195–212. doi: 10.1093/neuonc/noae041 (PMC11226873; doi:10.1093/neuonc/noae041)
Supplement: noae041_suppl_Supplementary_Data_S1 [file noae041_suppl_supplementary_data_s1.docx]

**Evidence Table:** ARS Appropriate Use Criteria for the Management of Brain Metastases in EGFR-mutated and ALK-fusion positive Non-Small Cell Lung Cancer

| **Reference** | **Study Type** | **Study Objective** | **Study Synopsis/ Results** | **Level of Evidence** |
| --- | --- | --- | --- | --- |
| Arbour K et al. *Twice weekly pulse and daily continuous-dose erlotinib as initial treatment for patients with epidermal growth factor receptor-mutant lung cancers and brain metastases*. Cancer 2018. 124 (1): 105-109 | Phase 1 non-randomized | To test safety and feasibility of twice weekly erlotinib in EGFRm NSCLC patients with BrM | Phase 1 study with 19 pts naïve to EGFR agent and RT to brain - pulse/continuous erlotinib. Intracranial ORR 75% lasting beyond systemic progression. 10-month PFS. No RT arm | 2 |
| Cai L. et al. *A comparative analysis of EGFR mutation status in association with the efficacy of TKI in combination with WBRT/SRS/surgery plus chemotherapy in brain metastasis from non-small cell lung cancer*. 2014 JNO. | Retrospective | To investigate the efficacy of EGFR TKIs in patients receiving WBRT, SRS, or surgery in patients with NSCLC and associate w EGFR status. | 282pts with NSCLC (50%EGFRm) getting RT vs RT+TKI. RT+TKI resulted in much better OS (31.9 v. 17 months). EGFRm had better systemic control w TKI | 2 |
| Camidge et al. *Brigatinib vs Crizotinib in Patients With ALK Positive NSCLC*. NEJM 2018. | Randomized phase 3 | To determine the efficacy of brigatinib vs crizotinib in the treatment of ALK-fusion NSCLC | Phase 3 study in TKI-naïve patients with ALK fusion NSCLC that allowed BrM at enrollment. IC response was a secondary endpoint. ICR 78% brigatinib v. 29% crizotinib; PFS 67% v. 21%. No RT arm | 1 |
| Chen H et al. *Concurrent versus sequential whole brain radiotherapy and TKI in EGFR-mutated NSCLC patients with brain metastasis: A single institution retrospective analysis*. Medicine (Baltimore). 97(44): e13014, 2018. | Retrospective | To compare outcomes of patients receiving EGFR TKI alone or in combination with WBRT in patients with EGFRm NSCLC BrM | 105 pts; 39 TKI alone, 34 TKI+WBRT, 32 RT then TKI. iORR 66.7%, 85.3%, and 75%, respectively (P < .05). Median iPFS 6.8, 12.4, and 9.1 months, respectively (P < .05). mOS 16.9, 24.5, and 21.3 (P < .001) | 3 |
| Chen Y et al. *First-line epidermal growth factor receptor (EGFR)-tyrosine kinase inhibitor alone or with whole-brain radiotherapy for brain metastases in patients with EGFR-mutated lung adenocarcinoma.* Cancer Science. 107 (12): 1800-1805, 2016. | Retrospective | To compare outcomes of patients receiving EGFR TKI alone or in combination with WBRT in patients with EGFRm NSCLC BrM | 132 patients. 79 TKI alone v. 53 with concomitant WBRT. iORR and iTTP both significantly better for TKI/WBRT. However, OS was not significantly different (48.0 v. 41.1 months, P=0.74) | 2 |
| Costa D, et al. *Clinical Experience With Crizotinib in Patients With Advanced ALK-Rearranged Non-Small-Cell Lung Cancer and Brain Metastases*. 2015. JCO 33(17): 1881-1888. | Retrospective | To determine benefit of ALK-TKI crizotinib in patients with ALK NSCLC and BrM enrolled in PROFILE studies | Retrospective sub-analysis of PROFILE 1005 and 1007 studies. 109pts RT naïve BrM v. 166 w RT - naïve intracranial DCR 56%, TTP 7mos; RT DCR 62%, TTP 13.2mos | 2 |
| Doherty MK et al*. Treatment options for patients with brain metastases from EGFR/ALK-driven lung cancer.* Radiother Oncol. 2017 May 123(2):195-202. | Retrospective | To evaluate impact of first line WBRT v SRS v TKI alone in patients with BrM from EGFRm NSCLC | 184 pts with EGFR/ALK NSCLC BrM. 120 WBRT, 37 SRS, 27 TKI alone. mTTIP longer in WBRT 50.5 months than SRS or TKI groups at 12 and 15months (p=0.0038). No significant difference was seen in median OS: 21.6months in the WBRT group, 23.9months in the SRS group and 22.6months in the TKI group (p=0.67) | 3 |
| Du XJ et al*. Upfront Cranial Radiotherapy vs. EGFR Tyrosine Kinase Inhibitors Alone for the Treatment of Brain Metastases From Non-small-cell Lung Cancer: A Meta-Analysis of 1465 Patients.* Front Oncol. 8:603. 2018. | Meta-analysis | To evaluate the impact of first line RT versus deferral of RT for BrM in EGFRm NSCLC | 13 studies with 1456 pts; Upfront RT higher OS (HR = 0.78, 95% CI = 0.65-0.93, P = 0.005) than TKI alone. Upfront RT+TKI higher OS (HR = 0.71, 95% CI = 0.58-0.86, P = 0.0005). Upfront SRS longer OS (HR = 0.37, 95% CI = 0.26-0.54, P < 0.00001). | M |
| Fan Y et al. *EGFR-TKI therapy for patients with brain metastases from non-small-cell lung cancer: a pooled analysis of published data*. Onco Targets Ther 7:2075-84, 2014. | Meta-analysis | To assess outcomes in patients receiving EGFR TKIs for BrM (NON-selected) | 16 studies with 464 pts. 362/464 pts EGFR status unknown. Higher ORR in the EGFRm than in the unselected group (85.0% vs 45.1%); a similar trend was observed for the DCR (94.6% vs 71.3%) | M |
| Gerber NK et al. *Erlotinib versus radiation therapy for brain metastases in patients with EGFR-mutant lung adenocarcinoma*. Int J Radiat Oncol Biol Phys 89 (2):322-329, 2014. | Retrospective | To evaluate the impact of first line RT versus deferral of RT for BrM in EGFRm NSCLC | 110 pts EGFRm w new BrM - erlotinib only (63), WBRT only (11), WBRT then erlotinib(21), SRS(15). SRS group had best OS 64mos, WBRT and erlotinib no sig difference (24 v 16mo) OS but TTP longer with WBRT | 2 |
| He Z et al. *Comparing the Efficacy of Concurrent EGFR-TKI and Whole-Brain Radiotherapy vs EGFR-TKI Alone as a First-Line Therapy for Advanced EGFR-mutated Non-Small-Cell Lung Cancer With Brain Metastases: A Retrospective Cohort Study.* Cancer Manag Res. 2019 Mar 14; 11:2129-2138. | Retrospective | To compare efficacy of concurrent EGFR-TKIs and WBRT vs EGFR-TKI alone in patients with EGFR-mutant NSCLS with brain metastases | 104 patients; 56 TKI+WBRT and 48 TKI alone. Adding WBRT significantly improved median intracranial PFS (17.7 vs 11.0 months, P=0.015), but not OS (28.1 vs 24.0 months, P=0.756). | 2 |
| Huber RM et al. *Brigatinib in Crizotinib-Refractory ALK+ NSCLC: 2-year Follow-up on Systemic and Intracranial Outcomes in the Phase 2 ALTA Trial*. JTO 15(3):404-415, 2020. | Phase 2, Randomized | To evaluate efficacy of brigatinib in patients with BrM in crizotinib refractory ALK-NSCLC | 222 pts randomized to 2 dose levels of brigatinib. mOS 29.5 months (18.2-not reached) vs. 34.1 months (27.7-not reached). IRC-confirmed iORR in patients with measurable baseline brain lesions was 50% (13 of 26) versus 67% (12 of 18); median duration of intracranial response was 9.4 versus 16.6 months. IRC-assessed iPFS was 12.8 versus 18.4 months. No RT arm. | 1 |
| Iuchi T et al*. Phase II trial of gefitinib alone without radiation therapy for Japanese patients with brain metastases from EGFR-mutant lung adenocarcinoma*. Lung cancer 82:282-287, 2013. | Phase 2 prospective,  single arm | To determine the efficacy of gefitinib alone in EGFRm NSCLC with BrM | 41 pts treated with gefitinib alone (no RT); CR 31.7%, CR+PR 88%; results vary by exon deletion. mPFS 14.5 months (95% CI, 10.2-18.3 months) and mOS was 21.9 months (95% CI, 18.5-30.3 months). No RT arm. | 2 |
| Jiang et al. *EGFR TKIs plus WBRT Demonstrated No Survival Benefit Other Than That of TKIs Alone in Patients with NSCLC and EGFR Mutation and Brain Metastases*. 2016 JTO (11): 10 1718-1728. | Retrospective | To determine whether there is benefit to TKI + WBRT versus TKI alone | Retrospective review of 230 pts with EGFRm BrMs: WBRT+TKI (51, 30 in first line) vs. TKI only (116 pts, 91 in first line). Outcome demonstrated PFS same in both groups, but the OS was worse with WBRT. | 2 |
| Jiang T et al. *Radiotherapy plus EGFR TKIs in non-small cell lung cancer patients with brain metastases: an update meta-analysis*. 2016. Cancer Med. | Meta-analysis | To review the efficacy of TKI, brain radiotherapy, and chemotherapy for BrM in EGFRm NSCLC | 15 study meta-analysis with 1552 patients. TKI + RT improves the DCR over RT alone or RT plus chemo RR 1.48. TKI + RT also better TTP and OS but at the cost of increased tox. Overall concurrent w TKI therapy best | M |
| Ke S.B. et al. *Therapeutic Effect of First-line Epidermal Growth Factor Receptor Tyrosine Kinase Inhibitor (EGFR-TKI) Combined with Whole Brain Radiotherapy on Patients with EGFR Mutation-positive Lung Adenocarcinoma and Brain Metastases*. 2018 Curr Med Sci. 38 (6): 1062-1068. | Retrospective | To compare the effect of EGFR TKI versus EGFR TKI plus WBRT in patients with EGFRm NSCLC BrM | 139 pts with EGFRm BrM. First line TKI only (79) vs TKI+WBRT (60). iTTP longer in EGFR TKI+ WBRT (30.0 v 18.2months) but no difference in OS (48.0 vs. 41.1 months). Also, no difference in OS w symptomatic v asymptomatic mets or >3 v <3 BrM. | 2 |
| Lee J et al. *Cranial Irradiation for Patients with Epidermal Growth Factor Receptor (EGFR) Mutant Lung Cancer Who Have Brain Metastases in the Era of a New Generation of EGFR Inhibitors*. 2019. The Oncologist. | Retrospective | To evaluate the effect of immediate (upfront) WBRT or SRS in patients with EGFRm NSCLC | 198pts with EGFRm NSCLC w BrM. All patients received TKI with WBRT early (75), SRS early (21), delayed RT(27), no RT(75) - SRS group best OS 55.7months vs. 18-21months for other groups | 2 |
| Li C et al. *Upfront whole brain radiotherapy for multiple brain metastases in patients with EGFR-mutant lung Adenocarcinoma.* Cancer Management and Research. 11: 3433-3443, 2019. | Retrospective | To determine the optimal management for patients with EGFRm NSCLC with BrM at initial diagnosis | 195 patients with EGFRm NSCLC with BrM at initial dx; iORR in WBRT then TKI 82%, concurrent WBRT/TKI 64%, upfront TKI alone 63%; upfront WBRT (n=67) had longer OS than concurrent WBRT/TKI (36 vs 25 months; P=0.006). This group notes differential effect based on DS-GPA. | 2 |
| Li SH et al*. Response to afatinib in treatment-naïve patients with advanced mutant epidermal growth factor receptor lung adenocarcinoma with brain metastases*. Expert Rev Anticancer Ther. 18(1):81-89, 2018. | Retrospective | To evaluate afatinib monotherapy v. afatinib + WBRT in patients with newly dx EGFRm NSCLC BrM | 28 pts afatinib only vs WBRT/afatinib. Worse ECOG and more symptoms in WBRT group. iORR was not different (81.8% v 88.2%, but CR rate 63.6% afatinib alone versus 17.6% WBRT/afatinib group. No differences in mOS and TTP. | 3 |
| Lin CH et al*. Increased survival with the combination of stereotactic radiosurgery and gefitinib for non-small cell lung cancer brain metastasis patients: a nationwide study in Taiwan*. Radiat Oncol. 10:127, 2015. | Retrospective | To assess outcome of BrM from NSCLC (NON selected) treated by WBRT followed by SRS, gefitinib, or the combination of SRS and gefitinib | 23874 pts; 20241 WBRT, 3379 WBRT + gefitinib, 155 WBRT + SRS, and 99 WBRT+ gefitinib + SRS for 99 patients. The median OS from BrM dx for WBRT, WBRT+ gefitinib, WBRT+ GK, WBRT+ gefitinib + GK groups was 0.53, 1.01, 1.46, and 2.25 years, respectively (p < 0.0001). | 2 |
| Lin H, et al. *Efficacy and safety of antitumor agents plus radiotherapy compared with radiotherapy alone for brain metastases from lung cancer*. 2017. Molec and Clin Onc. | Meta-analysis | To investigate the safety and efficacy of different therapeutic regimens for lung CA BrM | 13 study meta-analysis with 1783 patients getting RT alone or RT with chemo, TKI, or endostatin. Drug plus RT better then RT only (HRs reported); ORR 1.38, TTP 0.71, PFS 0.6, OS 0.8 but G3 toxicity HR + 2.57 | M |
| Liu S. *Radiotherapy for asymptomatic brain metastasis in epidermal growth factor receptor mutant non-small cell lung cancer without prior tyrosine kinase inhibitors treatment: a retrospective clinical study*. 2015. Rad Onc. | Retrospective | To determine the appropriate timing of brain radiotherapy for asymptomatic BrM in EGFRm NSCLC | 96pts asymptomatic TKI naïve pts with EGFRm BrM. RT upfront (39), delayed RT (23), no RT(34) - RT timing was not associated with better OS or PFS | 2 |
| Liu Y et al. *Concurrent brain radiotherapy and EGFR-TKI may improve intracranial metastases control in non-small cell lung cancer and have survival benefit in patients with low DS-GPA score*. Oncotarget 2017. 98 (1): 235-236. | Retrospective | To determine timing of RT with TKI in patients with BrM from EFGRm NSCLC | Retrospective review of 113 pts EGFRm with TKI naïve BrM; TKI+RT (49) vs. TKI only (64). RT group has better PFS, but OS is not significantly different. This is most significant for GPA scores 0-2. | 2 |
| Magnuson WJ et al*. Impact of Deferring Radiation Therapy in Patients With Epidermal Growth Factor Receptor-Mutant Non-Small Cell Lung Cancer Who Develop Brain Metastases.* Int J Radiat Oncol Biol Phys*.* 95(2):673-9. 2016. | Retrospective | To evaluate the impact of first line RT versus deferral of RT for BrM in EGFRm NSCLC | 50 pts; 17 TKI then SRS or WBRT, 17 WBRT then TKI, 16 SRS then TKI. mOS longer if RT first vs. upfront EGFR-TKI (34.1 vs 19.4 months; P=.01). SRS group had longer OS vs. upfront EGFR-TKI group (58.4 vs 19.4 months; P=.01), but the WBRT group did not (29.9 vs 19.4 months; P=.09) | 3 |
| Magnuson, W.J. et al. *Management of Brain Metastases in Tyrosine Kinase Inhibitor-Naive Epidermal Growth Factor Receptor-Mutant Non-Small-Cell Lung Cancer: A Retrospective Multi-Institutional Analysi*s. 2017. JCO 35(10): 1070-1077 | Retrospective | To determine the optimal management for patients with EGFRm NSCLC and new BrM | 6 institutions, 351 patients with EGFRm NSCLC and new BrM. OS for the SRS (n = 100), WBRT (n = 120), and EGFR-TKI/Erlotinib (n = 131) cohorts was 46, 30, and 25 months, respectively (P < .001). | 2 |
| Miyawaki E et al. *Optimal Sequence of Local and EGFR-TKI Therapy for EGFR-mutant Non-small cell lung cancer with brain metastases stratified by number of brain metastases*. Int J Radiat Oncol Biol Phys 104:604-613, 2019. | Retrospective | To determine the optimal timing of SRS, surgery, and EGFR-TKI in patients with EGFRm BrM | 176 pts treated with TKI alone or SRS or WBRT with TKI and split by # of metastases. 61% TKI only, 39% RT+TKI (31SRS, 27WBRT, 10 surgery - divided by 1-4 vs 5mets). Local therapy better OS v.TKI (mOS 35 vs 23 months; hazard ratio, 0.54). There was no difference in OS between the local therapy and TKI groups for patients with ≥5 BMs. | 2 |
| *Ni J et al. Optimal Timing and Clinical Value of Radiotherapy in Advanced ALK-rearranged Non-Small Cell Lung Cancer With or Without Baseline Brain Metastases: Implications From Pattern of Failure Analyses.* 2019 Radiat Oncol. Mar 13;14(1):44. | Retrospective | To determine the pattern of failure and clinical value of radiotherapy in metastatic crizotinib-treated ALK-mutant lung cancer, with or without baseline brain metastases | 35 of 93 patients had baseline brain metastases; patients who had baseline brain metastases, and received brain radiotherapy had significant longer PFS (median 6.0 vs 13.5 months, p = 0.006) | 3 |
| Reunwetwattana T et al. *CNS response to Osimertinib versus Standard Epidermal Growth Factor Receptor Tyrosine Kinase Inhibitors in Patients with untreated EGFR-mutated advanced non-small cell lung cancer*. J Clin Oncol 36:3290-3297. | Randomized Phase 3 | To evaluate CNS responses to osimertinib versus 1st generation EGFR inhibitors in pts with untreated EGFRm NSCLC | CNS sub-group from FLAURA P3 RTC. 128 pts with brain scans at baseline. Median iPFS was not reached with osi (95% CI, 16.5 months-NC) and 13.9 months (95% CI, 8.3 months -NC) with standard EGFR-TKIs (hazard ratio, 0.48; 95% CI, 0.26 to 0.86; P = .014) CNS ORRs 91% and 68% in patients with ≥ one measurable CNS lesion (odds ratio, 4.6; 95% CI, 0.9 to 34.9; P = .066). No RT Arm. | 1 |
| Saruwatari K et al. *Upfront Cranial Radiotherapy Followed by Erlotinib Positively Affects Clinical Outcomes of Epidermal Growth Factor Receptor-mutant Non-small Cell Lung Cancer With Brain Metastases.* Anticancer Res. 39(2):923-931, *2019*. | Retrospective | To investigate optimal management for EGFR NSCLC patients with BrM. | 81 pts; 30 received upfront RT, 51 no RT; upfront RT longer PFS. Erlotinib and upfront CRT were independent predictive factors for overall survival (OS) (erlotinib: HR 0.21; 95% CI, 0.10-0.48; p<0.001; upfront CRT: HR 0.42; 95% CI, 0.20-0.88; p=0.022) | 2 |
| Soon YY et al. *EGFR Tyrosine Kinase Inhibitors Versus Cranial Radiation Therapy for EGFR Mutant Non-Small Cell Lung Cancer With Brain Metastases: A Systematic Review and Meta-Analysis.* Radiother Oncol. 2015 Feb;114(2):167-72 | Review/Meta-Analysis | To determine if upfront cranial radiotherapy improves intracranial disease control and survival outcomes in EGFR mutant NSCLC with brain metastases relative to TKIs alone | 363 patients in 12 studies. Upfront cranial radiotherapy results in similar overall intracranial disease response rate (0.93,95% CI 0.82–1.06; p = 0.53), improved 4-month intracranial disease PFS (RR 1.06, 95% CI 1.00–1.12; p = 0.03), improved two-year OS (RR 1.33, 95% CI 1.00–1.77; p = 0.05) but caused more neurological adverse events. | 2 |
| Sung SY et al. *Intracranial control and survival outcome of tyrosine kinase inhibitor (TKI) alone versus TKI plus radiotherapy for brain metastasis of epidermal growth factor receptor-mutant non-small cell lung cancer.* J Neurooncol 139(1):205-213. 2018. | Retrospective | To evaluate efficacy of TKIs with and without RT for BrM in EGFRm NSCLC | 81 pts TKI vs TKI/RT - RT group more symptoms, larger lesions. CR+PR 70.8% vs 87.5%, local failure 19.6% TKI vs 5%, distant failure 58.5% vs 45%, no diff in 2-year survival (52.7 vs 44.1%) or neurological death (15.8 vs 23.4%) | 3 |
| Wang C et al. *Comparison of up-front radiotherapy and TKI with TKI alone for NSCLC with brain metastases and EGFR mutation: A meta-analysis.* Lung Cancer. 122:94-44, 2018. | Meta-analysis | To evaluate efficacy of TKIs with and without RT for BrM in EGFRm NSCLC | 7 studies with 1086 pts; upfront RT better than TKI alone iPFS HR=0.72 and OS HR=0.70. RT better if 1-3 BrM vs.4+ BrM HR=0.54 | M |
| Wang C et al. *The Efficacy of Upfront Intracranial Radiation with TKI Compared to TKI Alone in the NSCLC Patients Harboring EGFR Mutation and Brain Metastases*. 2019. J Cancer. 10(9): 1985-1990. | Retrospective | To determine timing of RT with TKI in patients with BrM from EFGRm NSCLC | 93 TKI naïve patients with EGFR NSCLC BrM RT w TKI (53) vs. TKI only (40). Risk of iPFS lower in RT w TKI group. HR 0.38. However, no difference in OS (35.4 v 35.8 months) | 2 |
| Wang W. et al. *Efficacy of brain radiotherapy plus EGFR-TKI for EGFR-mutated non-small cell lung cancer patients who develop brain metastasis*. 2018. Arch Med Sci 14 (6): 1298-1307. | Retrospective | To determine the appropriate timing of brain radiotherapy and EGFR TKI for BrM in EGFRm NSCLC | 181 patients with EGFRm NSCLC BrM. 49 patients w symptomatic mets had better mOS if they had SRS v WBRT (37.7 v. 21.1 month). Asymptomatic pts had better median OS with SRS first than TKI only first (24.9 vs. 17.4month). Median OS concurrent RT/TKI (21.9month)>RT then TKI (26.2month)>TKI alone(17.1month) was not statistically different | 2 |
| Wu Y. *CNS efficacy of osimertinib in patients with T790M-positive advanced non-small-cell lung cancer: data from a randomized Phase III trial (Aura3)* 2018. JCO 36(26): 2702-2709. | Randomized phase 3 | To determine the efficacy of osimertinib vs. platinum-pemetrexed in EGFRm NSCLC; CNS subset analysis of AURA3 | AURA3 subset analysis; EGFRm NSCLC failing first line TKI; 46pts w measurable CNS disease - ORR 70% osi vs 31% plat-pemetrexed. No RT arm | 1 |
| Yang JJ et al. *Icotinib versus whole-brain irradiation in patients with EGFR-mutant non-small-cell lung cancer and multiple brain metastases (BRAIN): a multicentre, phase 3, open-label, parallel, randomized controlled trial*. 2017 | Randomized phase 3 | To compare the efficacy of icotinib alone to WBRT +/- chemotherapy in patients with EGFRm NSCLC BrM | 176 pts randomized 1:1 (85 TKI, 91 WBRT, 18 withdrew after being assigned WBRT). iPFS 10 months TKI v 4.8 months WBRT HR 0.56 p=0.014. No difference in OS (18 months TKI v 20.1 months WBRT, p=0.73). | 1 |
| Zhang Z et al. *Anaplastic lymphoma kinase inhibitors in non-small cell lung cancer patients with brain metastases: a meta-analysis*. J Thorac Disease. 2019. 11(4):1397-1409. | Meta-analysis | To determine the efficacy of ALK inhibitors in BrM from ALK-fusion NSCLC | Meta-analysis of 20 studies with 2715 pts across 4 ALK inhibitors. Pooled iORR 48%. iORR 79% alectinib, 45% ceritinib, 48% brigatinib, 18% crizotinib. No RT arm | M |
| Zheng Hong, et al*. Clinical outcomes of WBRT plus EGFR-TKIs versus WBRT or TKIs alone for the treatment of cerebral metastatic NSCLC patients: A meta-analysis*. Oncotarget 8:57356-57364, 2017. | Meta-analysis | To determine whether there is benefit to TKI + WBRT versus TKI alone | 1041 pts (9 retrospective, 1RCT) WBRT/TKI vs WBRT only vs TKI only; OS better in EGFRm patients with TKI only compared to WBRT/TKI HR 1.25 p=0.08, but CI is 0.95-2.15. iPFS HR 1.3 p=0.03 in favor of TKI alone. | M |
| Zhu, Q et al. *Clinical outcome of tyrosine kinase inhibitors alone or combined with radiotherapy for brain metastases from epidermal growth factor receptor (EGFR) mutant non-small cell lung cancer (NSCLC)*. Oncotarget. 2017 Feb 21;8(8):13304-13311. | Retrospective | To compare outcomes between TKI monotherapy and TKI combined with brain radiotherapy for NSCLC patients with brain metastasis | 133 patients, 67 with TKI+RT and 66 with TKI alone; median cranial PFS of 16.0 months and 11.5 months, respectively in favor of TKI+RT (p=0.017); mOS of 22 months and 15 months, respectively in favor of TKI+RT (p=0.015). On multivariate analysis: treatment with TKI + RT (P = 0.012, HR=1.888 [1.150,3.100]) and intracranial metastasis alone (P = 0.037, HR = 1.807[1.038,3.148]) were associated with longer OS | 2 |
| Zhuang H et al*. Phase II study of whole brain radiotherapy with or without erlotinib in patients with multiple brain metastases from lung adenocarcinoma.* Drug Des Devel Ther.7:1179-86. 2013. | Phase 2 cohort study,  non-randomized | To compare outcomes of patients receiving EGFR TKI alone or in combination with WBRT in patients with NSCLC BrM (EGFR NON-selected) | No benefit or disadvantage to WBRT/TKI in EGFR-non-tested population | 2 |
