# Supplementary material for: Executive summary of the American Radium Society appropriate use criteria for brain metastases in epidermal growth factor receptor mutated-mutated and ALK-fusion non-small cell lung cancer
Source: Neuro Oncol. 2024 Mar 9;26(7):1195–212. doi: 10.1093/neuonc/noae041 (PMC11226873; doi:10.1093/neuonc/noae041)
Supplement: noae041_suppl_Supplementary_Data_S2 [file noae041_suppl_supplementary_data_s2.docx]

**ARS Voting Tables for Brain Metastases from EGFRm and ALK+ NSCLC**

*WBRT= for purposes of the cases presented here, WBRT administered with memantine and with hippocampal avoidance when appropriate.

**CASE 1 (a-d)**

Clinical Condition: Newly diagnosed NSCLC with targetable mutation (EGFR or ALK) with multiple asymptomatic brain metastases

Age 62

KPS 80

Extracranial disease Newly diagnosed, untreated lung + liver involvement

Size of brain metastases all 4 mm maximum diameter, no significant edema

Systemic therapy planned 3^rd^ generation CNS penetrant EGFR or ALK TKI

**Variant 1a**:

# of brain metastases 2-4

| **Treatment** | **Rating Category** | **Final Tabulations** | | | | | | | | | **Group Median Rating** | **Disagree** |
| --- | --- | --- | --- | --- | --- | --- | --- | --- | --- | --- | --- | --- |
|  |  | **1** | **2** | **3** | **4** | **5** | **6** | **7** | **8** | **9** |  |  |
| **No Radiation, TKI alone** | M* | 1 |  |  |  | 4 | 1 | 4 | 1 | 1 | 5* | X |
| **SRS to all metastases and TKI** | A |  |  |  |  |  |  | 2 | 7 | 4 | 8 |  |
| **SRS to selected metastases, and TKI** | M* | 1 |  | 2 |  | 2 | 2 | 3 | 1 | 1 | 5* | X |
| **WBRT * then TKI** | U | 4 | 5 | 1 | 2 |  |  |  |  |  | 2 |  |
| **WBRT* with concurrent TKI followed by TKI** | U | 6 | 4 |  | 2 |  |  |  |  |  | 1.5 |  |
| 1. **Rating**: **A**-Usually appropriate; **M**-May be appropriate; **U**-Usually not appropriate 2. **Per the UCLA/RAND Appropriateness Method** - * Disagreement, i.e., the variation of the individual ratings from the median rating indicates panel disagreement on the final recommendation (see narrative text). Group median rating is set automatically to 5. | | | | | | | | | | | | |

**Variant 1b**:

# of brain metastases 5-10

| **Treatment** | **Rating Category** | **Final Tabulations** | | | | | | | | | **Group Median Rating** | **Disagree** |
| --- | --- | --- | --- | --- | --- | --- | --- | --- | --- | --- | --- | --- |
|  |  | **1** | **2** | **3** | **4** | **5** | **6** | **7** | **8** | **9** |  |  |
| **No Radiation, TKI alone** | M* |  | 1 |  |  | 3 |  | 5 | 1 | 2 | 5* | X |
| **SRS to all metastases and TKI** | A |  |  |  |  |  | 2 | 5 | 4 | 1 | 7 |  |
| **SRS to selected metastases, and TKI** | M* |  |  |  | 1 | 3 | 3 | 1 | 2 | 2 | 5* | X |
| **WBRT * then TKI** | M* | 1 | 1 | 1 | 2 | 2 | 2 | 2 | 1 |  | 5* | X |
| **WBRT* with concurrent TKI followed by TKI** | M* | 1 | 1 | 2 | 3 | 1 | 2 | 1 | 1 |  | 5* | X |
| 1. **Rating**: **A**-Usually appropriate; **M**-May be appropriate; **U**-Usually not appropriate 2. **Per the UCLA/RAND Appropriateness Method** - * Disagreement, i.e., the variation of the individual ratings from the median rating indicates panel disagreement on the final recommendation (see narrative text). Group median rating is set automatically to 5. | | | | | | | | | | | | |

**Variant 1c**:

# of brain metastases 11-20

| **Treatment** | **Rating Category** | **Final Tabulations** | | | | | | | | | **Group Median Rating** | **Disagree** |
| --- | --- | --- | --- | --- | --- | --- | --- | --- | --- | --- | --- | --- |
|  |  | **1** | **2** | **3** | **4** | **5** | **6** | **7** | **8** | **9** |  |  |
| **No Radiation, TKI alone** | M* |  |  |  | 1 |  | 3 | 4 | 3 | 1 | 5* | X |
| **SRS to selected metastases, and TKI** | M |  |  |  | 2 | 4 | 3 | 1 | 2 |  | 5.5 |  |
| **WBRT * then TKI** | M* |  |  |  | 1 | 1 | 5 | 3 | 2 |  | 5* | X |
| **WBRT* with concurrent TKI followed by TKI** | M* |  |  | 1 | 1 | 5 | 1 | 2 | 2 |  | 5* | X |
| 1. **Rating**: **A**-Usually appropriate; **M**-May be appropriate; **U**-Usually not appropriate 2. **Per the UCLA/RAND Appropriateness Method** - * Disagreement, i.e., the variation of the individual ratings from the median rating indicates panel disagreement on the final recommendation (see narrative text). Group median rating is set automatically to 5. | | | | | | | | | | | | |

**Variant 1d**:

# of brain metastases >20

| **Treatment** | **Rating Category** | **Final Tabulations** | | | | | | | | | **Group Median Rating** | **Disagree** |
| --- | --- | --- | --- | --- | --- | --- | --- | --- | --- | --- | --- | --- |
|  |  | **1** | **2** | **3** | **4** | **5** | **6** | **7** | **8** | **9** |  |  |
| **No Radiation, TKI alone** | M* |  |  |  | 1 |  | 3 | 5 | 3 |  | 5* | X |
| **SRS to all metastases and TKI** | M* |  | 1 | 4 | 2 | 3 |  |  | 2 |  | 5* | X |
| **SRS to selected metastases, and TKI** | M |  | 2 | 1 | 3 | 4 | 2 |  |  |  | 4.5 |  |
| **WBRT * then TKI** | M* |  |  |  | 1 | 3 | 2 | 5 |  | 1 | 5* | X |
| **WBRT* with concurrent TKI followed by TKI** | M* |  |  |  | 2 | 6 |  | 3 |  | 1 | 5* | X |
| 1. **Rating**: **A**-Usually appropriate; **M**-May be appropriate; **U**-Usually not appropriate 2. **Per the UCLA/RAND Appropriateness Method** - * Disagreement, i.e., the variation of the individual ratings from the median rating indicates panel disagreement on the final recommendation (see narrative text). Group median rating is set automatically to 5. | | | | | | | | | | | | |

**CASE 2 (a-d)**

Clinical Condition: NSCLC with a targetable mutation (EGFR or ALK) with systemic progression on a 1^st^ or 2^nd^ generation TKI with multiple new asymptomatic brain metastases

Age 54

KPS 80

Extracranial disease Progressive bone disease after 1^st^/2^nd^ generation TKI

Size of brain metastases All 4 mm maximum diameter, minimal edema

Systemic therapy plan 3^rd^ generation EGFR or ALK TKI

**Variant 2a**:

# of brain metastases 2-4

| **Treatment** | **Rating Category** | **Final Tabulations** | | | | | | | | | **Group Median Rating** | **Disagree** |
| --- | --- | --- | --- | --- | --- | --- | --- | --- | --- | --- | --- | --- |
|  |  | **1** | **2** | **3** | **4** | **5** | **6** | **7** | **8** | **9** |  |  |
| **No Radiation, TKI alone** | M* | 1 |  |  | 1 | 2 | 3 | 5 |  |  | 5* | X |
| **SRS to all metastases and TKI** | A |  |  |  |  |  | 1 | 2 | 6 | 4 | 8 |  |
| **SRS to selected metastases, and TKI** | M* | 1 |  | 1 | 1 |  | 3 | 4 | 1 | 1 | 5* | X |
| **WBRT * then TKI** | U | 5 | 3 | 3 | 1 |  | 1 |  |  |  | 2 |  |
| **WBRT* with concurrent TKI followed by TKI** | U | 6 | 3 | 2 | 1 |  |  | 1 |  |  | 2 |  |
| 1. **Rating**: **A**-Usually appropriate; **M**-May be appropriate; **U**-Usually not appropriate 2. **Per the UCLA/RAND Appropriateness Method** - * Disagreement, i.e., the variation of the individual ratings from the median rating indicates panel disagreement on the final recommendation (see narrative text). Group median rating is set automatically to 5. | | | | | | | | | | | | |

**Variant 2b**:

# of brain metastases 5-10

| **Treatment** | **Rating Category** | **Final Tabulations** | | | | | | | | | **Group Median Rating** | **Disagree** |
| --- | --- | --- | --- | --- | --- | --- | --- | --- | --- | --- | --- | --- |
|  |  | **1** | **2** | **3** | **4** | **5** | **6** | **7** | **8** | **9** |  |  |
| **No Radiation, TKI alone** | M* |  | 1 |  | 1 | 2 | 4 | 4 |  |  | 5* | X |
| **SRS to all metastases and TKI** | A |  |  |  |  |  | 2 | 3 | 4 | 4 | 8 |  |
| **SRS to selected metastases, and TKI** | M* |  | 1 |  | 1 | 1 | 5 | 2 | 1 | 1 | 5* | X |
| **WBRT * then TKI** | M* |  | 1 | 1 | 2 | 2 | 3 | 2 | 1 |  | 5* | X |
| **WBRT* with concurrent TKI followed by TKI** | M* |  | 1 | 2 | 3 | 2 | 2 | 1 | 1 |  | 5* | X |
| 1. **Rating**: **A**-Usually appropriate; **M**-May be appropriate; **U**-Usually not appropriate 2. **Per the UCLA/RAND Appropriateness Method** - * Disagreement, i.e., the variation of the individual ratings from the median rating indicates panel disagreement on the final recommendation (see narrative text). Group median rating is set automatically to 5. | | | | | | | | | | | | |

**Variant 2c**:

# of brain metastases 11-20

| **Treatment** | **Rating Category** | **Final Tabulations** | | | | | | | | | **Group Median Rating** | **Disagree** |
| --- | --- | --- | --- | --- | --- | --- | --- | --- | --- | --- | --- | --- |
|  |  | **1** | **2** | **3** | **4** | **5** | **6** | **7** | **8** | **9** |  |  |
| **No Radiation, TKI alone** | M |  |  |  | 1 | 3 | 5 | 2 | 1 |  | 6 |  |
| **SRS to all metastases and TKI** | M* |  |  | 1 | 2 | 3 | 2 | 3 | 1 |  | 5* | X |
| **SRS to selected metastases, and TKI** | M |  |  |  | 2 | 4 | 3 | 2 | 1 |  | 5.5 |  |
| **WBRT * then TKI** | M* |  |  |  | 1 | 5 |  | 4 | 2 |  | 5* | X |
| **WBRT* with concurrent TKI followed by TKI** | M |  | 1 | 1 | 5 | 4 |  | 1 |  |  | 4 |  |
| 1. **Rating**: **A**-Usually appropriate; **M**-May be appropriate; **U**-Usually not appropriate 2. **Per the UCLA/RAND Appropriateness Method** - * Disagreement, i.e., the variation of the individual ratings from the median rating indicates panel disagreement on the final recommendation (see narrative text). Group median rating is set automatically to 5. | | | | | | | | | | | | |

**Variant 2d**:

# of brain metastases >20

| **Treatment** | **Rating Category** | **Final Tabulations** | | | | | | | | | **Group Median Rating** | **Disagree** |
| --- | --- | --- | --- | --- | --- | --- | --- | --- | --- | --- | --- | --- |
|  |  | **1** | **2** | **3** | **4** | **5** | **6** | **7** | **8** | **9** |  |  |
| **No Radiation, TKI alone** | M* |  |  |  | 1 | 4 | 3 | 2 | 2 |  | 5* | X |
| **SRS to all metastases and TKI** | M* |  |  | 4 | 3 | 2 | 2 |  | 1 |  | 5* | X |
| **SRS to selected metastases, and TKI** | M | 1 | 1 |  | 1 | 8 |  | 1 |  |  | 5 |  |
| **WBRT * then TKI** | A |  | 1 |  |  | 1 | 1 | 4 | 2 | 4 | 7 |  |
| **WBRT* with concurrent TKI followed by TKI** | M* |  |  |  | 1 | 5 | 1 | 3 | 2 |  | 5* | X |
| 1. **Rating**: **A**-Usually appropriate; **M**-May be appropriate; **U**-Usually not appropriate 2. **Per the UCLA/RAND Appropriateness Method** - * Disagreement, i.e., the variation of the individual ratings from the median rating indicates panel disagreement on the final recommendation (see narrative text). Group median rating is set automatically to 5. | | | | | | | | | | | | |

**CASE 3** Newly diagnosed NSCLC with targetable mutation (EGFR or ALK) with 2 symptomatic, unresectable brain metastases

Age 50

KPS 70

Extracranial disease newly diagnosed; lung and adrenal gland metastases

# of brain metastases 2

Size of brain metastases 1.3 cm (pons) and 4 mm (left frontal)

Systemic therapy CNS penetrant 3^rd^ generation TKI planned

| **Treatment** | **Rating Category** | **Final Tabulations** | | | | | | | | | **Group Median Rating** | **Disagree** |
| --- | --- | --- | --- | --- | --- | --- | --- | --- | --- | --- | --- | --- |
|  |  | **1** | **2** | **3** | **4** | **5** | **6** | **7** | **8** | **9** |  |  |
| **No Radiation, TKI alone** | M* | 1 | 1 | 2 | 2 | 4 | 2 |  |  |  | 5* | X |
| **WBRT* followed by TKI** | M | 1 |  | 1 | 3 | 4 | 3 |  |  |  | 5 |  |
| **WBRT* concurrent with TKI** | U | 3 | 2 | 5 | 1 | 1 | 1 |  |  |  | 3 |  |
| **WBRT* with planned SRS boost, followed by TKI** | M* | 1 |  | 4 | 1 | 3 | 2 | 1 |  |  | 5* | X |
| **WBRT* with planned SRS boost and concurrent TKI** | M* | 1 |  | 5 | 3 | 3 |  |  |  |  | 5* | X |
| **SRS alone for both lesions^a^; TKI to follow** | A |  |  |  |  |  |  | 8 | 2 | 3 | 7 |  |
| 1. **Rating**: **A**-Usually appropriate; **M**-May be appropriate; **U**-Usually not appropriate 2. **Per the UCLA/RAND Appropriateness Method** - * Disagreement, i.e., the variation of the individual ratings from the median rating indicates panel disagreement on the final recommendation (see narrative text). Group median rating is set automatically to 5. | | | | | | | | | | | | |

^a^Fractionation at the discretion of the treating radiation oncologist

**Case 4** Newly diagnosed NSCLC with targetable mutation (EGFR or ALK) with multiple brain metastases of larger size and symptoms at time of diagnosis

Age 50

KPS 70

Extracranial disease New diagnosis; lung and liver involvement

# of brain metastases 4 (mildly symptomatic from the L frontal metastases)

Size of brain metastases Maximum diameter 3.5 cm, 1.5 cm, 0.8 cm, & 0.6 cm.

Location of lesions Left frontal (3.5 cm), right temporal (1.5cm), left parietal lesion (0.8 cm), left cerebellar (0.6 cm)

Systemic therapy CNS penetrant 3^rd^ generation TKI planned

| **Treatment** | **Rating Category** | **Final Tabulations** | | | | | | | | | **Group Median Rating** | **Disagree** |
| --- | --- | --- | --- | --- | --- | --- | --- | --- | --- | --- | --- | --- |
|  |  | **1** | **2** | **3** | **4** | **5** | **6** | **7** | **8** | **9** |  |  |
| **No Radiation, TKI alone** | M* | 1 |  | 4 | 2 | 3 | 1 | 1 |  |  | 5* | X |
| **WBRT* followed by TKI** | M* | 1 |  | 2 | 2 | 3 | 2 | 1 | 1 |  | 5* | X |
| **WBRT* concurrent with TKI** | M* | 1 |  | 3 | 3 | 2 | 1 | 1 | 1 |  | 5* | X |
| **WBRT* with planned SRS boost, followed by TKI** | M* | 1 |  | 4 | 1 | 1 | 2 | 2 | 1 |  | 5* | X |
| **WBRT* with planned SRS boost and concurrent TKI** | M* | 1 |  | 6 | 2 |  | 1 | 1 |  |  | 5* | X |
| **SRS alone for all lesions^a^; TKI to follow** | A |  |  | 1 |  | 1 | 1 | 6 | 1 | 2 | 7 |  |
| **Resection of left frontal lesion 🡪WBRT* followed by TKI** | M* | 1 |  | 1 | 1 | 1 | 4 | 3 | 1 |  | 5* | X |
| **Resection of left frontal lesion 🡪WBRT* with concurrent TKI** | M* | 1 |  | 2 | 3 | 1 | 2 | 2 | 1 |  | 5* | X |
| **Resection of left frontal lesion 🡪 SRS to cavity and intact lesions (single or multi-fraction) followed by TKI** | A |  |  |  |  |  |  | 5 | 4 | 4 | 8 |  |
| **Pre-operative SRS of lesions->resection of the L frontal lesion ->TKI** | M* |  |  |  |  | 3 | 2 | 5 | 2 |  | 5* | X |
| 1. **Rating**: **A**-Usually appropriate; **M**-May be appropriate; **U**-Usually not appropriate 2. **Per the UCLA/RAND Appropriateness Method** - * Disagreement, i.e., the variation of the individual ratings from the median rating indicates panel disagreement on the final recommendation (see narrative text). Group median rating is set automatically to 5. | | | | | | | | | | | | |

**CASE 5 (a-d)** Isolated CNS progression in NSCLC with targetable mutation (EGFR or ALK) while on 3^rd^ generation CNS penetrant drug. *Variants presented here are based on mutational profile.*

Age 50

KPS 80

Extracranial disease stable disease

# of brain metastases 7

Size of brain metastases all 4-6 mm maximum diameter, non-eloquent brain

Systemic therapy plan pending your consultation

**Variant 5a:**

Patient declines brain biopsy or CSF testing to define mutational profile.

| **Treatment** | **Rating Category** | **Final Tabulations** | | | | | | | | | **Group Median Rating** | **Disagree** |
| --- | --- | --- | --- | --- | --- | --- | --- | --- | --- | --- | --- | --- |
|  |  | **1** | **2** | **3** | **4** | **5** | **6** | **7** | **8** | **9** |  |  |
| **No Radiation, TKI alone at a higher dose^b^** | M |  | 1 |  | 1 | 5 | 3 | 2 |  |  | 5 |  |
| **No Radiation, switch to a pemetrexed based regimen** | M | 1 |  |  | 1 | 5 | 3 | 1 | 1 |  | 5 |  |
| **SRS for all metastases; continue TKI at standard dose^c^** | A |  |  |  |  |  |  | 6 | 4 | 3 | 8 |  |
| **SRS for all metastases; followed by higher dose of TKI^b^** | A |  |  |  | 1 |  |  | 5 | 5 | 2 | 8 |  |
| **SRS for all metastases; followed by a pemetrexed based regimen** | M* |  |  |  |  | 1 | 3 | 4 | 3 | 1 | 5* | X |
| **WBRT* followed by TKI at standard dose^c^** | M | 1 |  | 1 | 3 | 3 | 4 |  |  |  | 5 |  |
| **WBRT* with concurrent standard dose^c^ TKI w standard dose^c^ TKI to follow** | M | 1 |  | 2 | 4 | 4 | 1 |  |  |  | 4 |  |
| **WBRT* switch to a pemetrexed based regimen** | M |  |  | 1 |  | 6 | 3 | 1 | 1 |  | 5 |  |
| 1. **Rating**: **A**-Usually appropriate; **M**-May be appropriate; **U**-Usually not appropriate 2. **Per the UCLA/RAND Appropriateness Method** - * Disagreement, i.e., the variation of the individual ratings from the median rating indicates panel disagreement on the final recommendation (see narrative text). Group median rating is set automatically to 5. | | | | | | | | | | | | |

^b^ i.e. 160mg daily osimertinib or 900mg BID alectinib

^c^ i.e. 80mg daily osimertinib or 600mg BID alectinib

**Variant 5b:**

Patient with T790M mutation on osimertinib undergoes brain biopsy or CSF liquid biopsy indicating a new MET exon 14 skipping alteration (EGFR TKI resistance mechanism, responsive to selumetinib)

| **Treatment** | **Rating Category** | **Final Tabulations** | | | | | | | | | **Group Median Rating** | **Disagree** |
| --- | --- | --- | --- | --- | --- | --- | --- | --- | --- | --- | --- | --- |
|  |  | **1** | **2** | **3** | **4** | **5** | **6** | **7** | **8** | **9** |  |  |
| **No radiation, osimertinib at higher dose^d^** | M |  | 1 | 1 | 2 | 5 | 3 |  |  |  | 5 |  |
| **No radiation, switch to selumetinib** | M |  |  |  | 1 | 6 | 2 | 2 | 1 |  | 5 |  |
| **No Radiation, selumetinib/osimertinib combination** | M |  | 1 |  | 3 | 4 | 2 | 2 |  |  | 5 |  |
| **No Radiation, switch to a pemetrexed based regimen** | M |  |  |  | 2 | 5 | 5 |  |  |  | 5 |  |
| **SRS for all metastases; continue osimertinib at standard dose^c^** | M |  |  | 1 | 2 | 2 | 1 | 6 |  |  | 5* | X |
| **SRS for all metastases; followed by higher dose of osimertinib^b^** | A |  | 1 | 1 |  | 1 |  | 6 | 3 |  | 7 |  |
| **SRS for all metastases; then selumetinib** | A | 1 |  |  |  | 1 | 1 | 7 | 2 | 1 | 7 |  |
| **SRS for all metastases; then selumetinib/osimertinib combination** | M* |  |  |  |  | 3 | 1 | 7 |  | 1 | 5* | X |
| **SRS for all metastases; followed by a pemetrexed based regimen** | M* |  |  |  |  | 5 | 1 | 6 |  |  | 5* | X |
| **WBRT* followed by Osimertinib at standard dose^c^** | M* | 1 |  | 3 |  | 3 | 3 | 2 |  |  | 5* | X |
| **WBRT* followed by Osimertinib at higher dose^b^** | M* | 1 |  | 2 |  | 3 | 4 | 2 |  |  | 5* | X |
| **WBRT* with concurrent and adjuvant Osimertinib at standard dose** | M* | 1 |  | 4 | 1 | 1 | 3 | 2 |  |  | 5* | X |
| **WBRT* with concurrent Osimertinib at standard dose followed by high dose osimertinib** | M* | 1 |  | 4 |  | 2 | 3 | 2 |  |  | 5* | X |
| **WBRT* followed by selumetinib** | M* | 1 |  | 1 |  | 4 | 3 | 3 |  |  | 5* | X |
| **WBRT* followed by selumetinib/osimertinib combination** | M | 1 |  | 1 |  | 7 | 2 | 1 |  |  | 5 |  |
| **WBRT* then switch to a pemetrexed based regimen** | M* | 1 |  | 2 |  | 4 | 4 | 1 |  |  | 5* | X |
| 1. **Rating**: **A**-Usually appropriate; **M**-May be appropriate; **U**-Usually not appropriate 2. **Per the UCLA/RAND Appropriateness Method** - * Disagreement, i.e., the variation of the individual ratings from the median rating indicates panel disagreement on the final recommendation (see narrative text). Group median rating is set automatically to 5. | | | | | | | | | | | | |

^b^ i.e. 160mg daily osimertinib or 900mg BID alectinib

^c^ i.e. 80mg daily osimertinib or 600mg BID alectinib

^d^ i.e. pulse dose erlotinib (1500mg q7 days) or pulse-daily dose erlotinib (1200mg two days a week, 50mg the other 5 days)

**Variant 5c:**

Patient undergoes brain biopsy or CSF liquid biopsy indicating a novel resistance mechanism

| **Treatment** | **Rating Category** | **Final Tabulations** | | | | | | | | | **Group Median Rating** | **Disagree** |
| --- | --- | --- | --- | --- | --- | --- | --- | --- | --- | --- | --- | --- |
|  |  | **1** | **2** | **3** | **4** | **5** | **6** | **7** | **8** | **9** |  |  |
| **No Radiation, TKI alone at a higher dose^b^** | M | 1 |  | 2 | 4 | 3 | 2 |  |  |  | 4 |  |
| **No Radiation, switch to a pemetrexed based regimen** | M | 1 |  |  | 1 | 5 | 5 |  |  |  | 5 |  |
| **SRS for all metastases; continue TKI at standard dose^c^** | M* |  |  |  |  | 2 | 3 | 5 | 2 |  | 5* | X |
| **SRS for all metastases; followed by higher dose of TKI^b^** | M* |  |  | 1 |  | 1 | 2 | 3 | 5 |  | 5* | X |
| **SRS for all metastases; followed by a pemetrexed based regimen** | A |  | 1 |  |  |  | 2 | 6 | 3 | 1 | 7 |  |
| **WBRT* followed by TKI at standard dose^c^** | M* |  | 1 | 1 | 2 | 3 | 3 | 2 |  |  | 5* | X |
| **WBRT* with concurrent standard dose TKI^c^ w standard dose TKI^c^ to follow** | M* |  | 1 | 3 | 1 | 3 | 2 | 2 |  |  | 5* | X |
| **WBRT* switch to a pemetrexed based regimen** | M |  | 1 |  | 1 | 4 | 4 | 2 |  |  | 5.5 |  |
| 1. **Rating**: **A**-Usually appropriate; **M**-May be appropriate; **U**-Usually not appropriate 2. **Per the UCLA/RAND Appropriateness Method** - * Disagreement, i.e., the variation of the individual ratings from the median rating indicates panel disagreement on the final recommendation (see narrative text). Group median rating is set automatically to 5. | | | | | | | | | | | | |

^b^ i.e. 160mg daily osimertinib or 900mg BID alectinib

^c^ i.e. 80mg daily osimertinib or 600mg BID alectinib

^d^ i.e. pulse dose erlotinib (1500mg q7 days) or pulse-daily dose erlotinib (1200mg two days a week, 50mg the other 5 days)

**Variant 5d:**

Patient undergoes brain biopsy or CSF liquid biopsy that retains the initial mutational/fusional status of the original tumor

| **Treatment** | **Rating Category** | **Final Tabulations** | | | | | | | | | **Group Median Rating** | **Disagree** |
| --- | --- | --- | --- | --- | --- | --- | --- | --- | --- | --- | --- | --- |
|  |  | **1** | **2** | **3** | **4** | **5** | **6** | **7** | **8** | **9** |  |  |
| **No Radiation, TKI alone at a higher dose^b^** | M | 1 |  |  | 1 | 3 | 5 | 2 |  |  | 6 |  |
| **No Radiation, switch to a pemetrexed based regimen** | M | 1 |  |  | 1 | 3 | 6 | 1 |  |  | 6 |  |
| **SRS for all metastases; continue TKI at standard dose^c^** | A |  |  | 1 |  |  | 1 | 7 | 3 | 1 | 7 |  |
| **SRS for all metastases; followed by higher dose of TKI^b^** | A |  |  |  |  |  | 1 | 5 | 5 | 2 | 7.5 |  |
| **SRS for all metastases; followed by a pemetrexed based regimen** | A |  |  |  |  | 2 | 1 | 8 | 1 |  | 7 |  |
| **WBRT* followed by TKI at standard dose^c^** | M |  |  |  | 1 | 4 | 5 | 2 |  |  | 6 |  |
| **WBRT* with concurrent standard dose TKI^c^ w standard dose TKI^c^ to follow** | M* |  |  | 3 | 1 | 3 | 3 | 2 |  |  | 5* | X |
| **WBRT* switch to a pemetrexed based regimen** | M* |  |  | 1 |  | 5 | 3 | 3 |  |  | 5* | X |
| 1. **Rating**: **A**-Usually appropriate; **M**-May be appropriate; **U**-Usually not appropriate 2. **Per the UCLA/RAND Appropriateness Method** - * Disagreement, i.e., the variation of the individual ratings from the median rating indicates panel disagreement on the final recommendation (see narrative text). Group median rating is set automatically to 5. | | | | | | | | | | | | |

^b^ i.e. 160mg daily osimertinib or 900mg BID alectinib

^c^ i.e. 80mg daily Osimertinib or 600mg BID alectinib

^d^ i.e. pulse dose erlotinib (1500mg q7 days) or pulse-daily dose erlotinib (1200mg two days a week, 50mg the other 5 days)

**CASE 6** Newly diagnosed NSCLC with targetable mutation (EGFR or ALK) with an isolated, symptomatic surgically resectable brain metastasis

Age 61

KPS 80 (seizure; hemiparesis that improved with steroids)

Extracranial disease: new diagnosis with lung and bone involvement

# of brain metastases 1

Size of brain metastases 2 cm right parieto-occipital cortex lesion with extensive

edema

Systemic therapy plan 3^rd^ generation TKI with CNS activity

| **Treatment** | **Rating Category** | **Final Tabulations** | | | | | | | | | **Group Median Rating** | **Disagree** |
| --- | --- | --- | --- | --- | --- | --- | --- | --- | --- | --- | --- | --- |
|  |  | **1** | **2** | **3** | **4** | **5** | **6** | **7** | **8** | **9** |  |  |
| **No surgery, no radiation, TKI alone** | M* | 1 |  | 5 | 3 | 2 |  | 1 |  |  | 5* | X |
| **No surgery, SRS followed by TKI** | M* | 1 |  |  | 1 | 3 | 2 | 4 |  | 1 | 5* | X |
| **Surgery, no radiation, followed by TKI** | M | 1 |  | 1 | 2 | 1 | 6 | 1 |  |  | 6 |  |
| **Surgery, SRS, followed by TKI** | A |  |  |  |  | 1 |  | 5 | 4 | 3 | 7.75 |  |
| **Pre-operative SRS, surgery, followed by TKI** | A |  |  |  |  | 2 | 1 | 6 | 3 |  | 7 |  |
| 1. **Rating**: **A**-Usually appropriate; **M**-May be appropriate; **U**-Usually not appropriate 2. **Per the UCLA/RAND Appropriateness Method** - * Disagreement, i.e., the variation of the individual ratings from the median rating indicates panel disagreement on the final recommendation (see narrative text). Group median rating is set automatically to 5. | | | | | | | | | | | | |

**CASE 7 (a-d)** NSCLC with newly diagnosed leptomeningeal metastases with a targetable mutation

**Variant 7a:**

Age 48

KPS 90 (no CNS symptoms)

Extracranial disease: new diagnosis

# of brain metastases No parenchymal metastases, diffuse LM

Systemic therapy plan 3^rd^ generation TKI with CNS activity

| **Treatment** | **Rating Category** | **Final Tabulations** | | | | | | | | | **Group Median Rating** | **Disagree** |
| --- | --- | --- | --- | --- | --- | --- | --- | --- | --- | --- | --- | --- |
|  |  | **1** | **2** | **3** | **4** | **5** | **6** | **7** | **8** | **9** |  |  |
| **No radiation, TKI alone** | A |  |  |  |  | 2 | 1 | 5 | 2 | 2 | 7 |  |
| WBRT followed by TKI | M* |  |  |  | 1 | 3 | 1 | 5 | 2 |  | 5* | X |
| CSI followed by TKI | M* |  | 1 | 3 | 4 | 3 | 1 |  |  |  | 5* | X |
| Hospice/best supportive care | M* |  | 1 | 3 | 1 | 4 | 2 | 1 |  |  | 5* | X |
| 1. **Rating**: **A**-Usually appropriate; **M**-May be appropriate; **U**-Usually not appropriate 2. **Per the UCLA/RAND Appropriateness Method** - * Disagreement, i.e., the variation of the individual ratings from the median rating indicates panel disagreement on the final recommendation (see narrative text). Group median rating is set automatically to 5. | | | | | | | | | | | | |

**Variant 7b:**

Age 48

KPS 80 (episodic confusion, possible seizure)

Extracranial disease: 1^st^ progression on a 1^st^ or 2^nd^ generation TKI

# of brain metastases 4 parenchymal metastases (1cm), diffuse LM

Systemic therapy plan 3^rd^ generation TKI with CNS activity

| **Treatment** | **Rating Category** | **Final Tabulations** | | | | | | | | | **Group Median Rating** | **Disagree** |
| --- | --- | --- | --- | --- | --- | --- | --- | --- | --- | --- | --- | --- |
|  |  | **1** | **2** | **3** | **4** | **5** | **6** | **7** | **8** | **9** |  |  |
| **No radiation, TKI alone** | M |  |  |  | 2 | 4 | 3 | 2 | 1 |  | 5.5 |  |
| **SRS to parenchymal metastases followed by TKI** | M |  | 2 |  | 4 | 3 | 3 |  |  |  | 4.5 |  |
| **WBRT followed by TKI** | A |  |  |  | 2 |  | 1 | 5 | 3 | 1 | 7 |  |
| **WBRT + SRS boost followed by TKI** | M* |  |  | 3 |  | 5 | 2 | 2 |  |  | 5* | X |
| **WBRT with concurrent TKI** | M* |  |  | 2 |  | 5 | 2 | 2 |  | 1 | 5* | X |
| **CSI followed by TKI** | M* |  | 1 | 3 | 4 | 3 | 1 |  |  |  | 5* | X |
| **Hospice/best supportive care** | M |  |  | 1 | 4 | 2 | 3 | 2 |  |  | 5 |  |
| 1. **Rating**: **A**-Usually appropriate; **M**-May be appropriate; **U**-Usually not appropriate 2. **Per the UCLA/RAND Appropriateness Method** - * Disagreement, i.e., the variation of the individual ratings from the median rating indicates panel disagreement on the final recommendation (see narrative text). Group median rating is set automatically to 5. | | | | | | | | | | | | |

**Variant 7c:**

Age 75

KPS 60 (difficulty chewing and eating from CN5 involvement)

Extracranial disease: newly diagnosed

# of brain metastases No parenchymal metastases, diffuse LM

Systemic therapy plan 3^rd^ generation TKI with CNS activity

| **Treatment** | **Rating Category** | **Final Tabulations** | | | | | | | | | **Group Median Rating** | **Disagree** |
| --- | --- | --- | --- | --- | --- | --- | --- | --- | --- | --- | --- | --- |
|  |  | **1** | **2** | **3** | **4** | **5** | **6** | **7** | **8** | **9** |  |  |
| **No radiation, TKI alone** | M* |  |  |  | 2 | 3 | 3 | 3 | 1 |  | 5* | X |
| **Radiation to skull base followed by TKI** | M* | 1 |  |  |  |  | 6 | 2 | 3 |  | 5* | X |
| **WBRT followed by TKI** | A |  |  |  |  | 2 | 1 | 6 | 2 | 1 | 7 |  |
| **WBRT with concurrent TKI** | M* |  |  | 3 | 1 | 4 | 2 | 1 |  | 1 | 5* | X |
| **CSI followed by TKI** | M* |  | 1 | 5 | 3 | 3 |  |  |  |  | 5* | X |
| **Hospice/best supportive care** | M* |  |  | 1 | 3 | 3 | 2 | 3 |  |  | 5* | X |
| 1. **Rating**: **A**-Usually appropriate; **M**-May be appropriate; **U**-Usually not appropriate 2. **Per the UCLA/RAND Appropriateness Method** - * Disagreement, i.e., the variation of the individual ratings from the median rating indicates panel disagreement on the final recommendation (see narrative text). Group median rating is set automatically to 5. | | | | | | | | | | | | |

**Variant 7d:**

Age 75

KPS 60 (significant CNS symptoms)

Extracranial disease: stable on 3^rd^ generation TKI

# of brain metastases 15 parenchymal metastases (all <5mm), diffuse LM

Systemic therapy plan pending your consult

| **Treatment** | **Rating Category** | **Final Tabulations** | | | | | | | | | **Group Median Rating** | **Disagree** |
| --- | --- | --- | --- | --- | --- | --- | --- | --- | --- | --- | --- | --- |
|  |  | **1** | **2** | **3** | **4** | **5** | **6** | **7** | **8** | **9** |  |  |
| **No radiation, higher dose TKI alone** | M |  |  | 1 |  | 7 | 2 | 2 |  |  | 5 |  |
| **SRS followed by higher dose TKI** | U | 1 | 3 | 5 | 1 | 1 |  |  | 1 |  | 3 |  |
| **WBRT followed by TKI** | A |  |  | 1 |  | 1 | 1 | 2 | 5 | 2 | 8 |  |
| **WBRT with concurrent TKI** | M* |  |  | 4 |  | 1 | 3 | 2 | 2 |  | 5* | X |
| **CSI followed by TKI** | M* |  |  | 4 | 4 | 3 |  |  |  | 1 | 5* | X |
| **Pemetrexed based regimen** | M* | 1 |  | 1 |  | 5 | 2 | 3 |  |  | 5* | X |
| **Pemetrexed based regimen, continue TKI** | M* | 1 |  | 1 |  | 5 | 2 | 2 | 1 |  | 5* | X |
| **Hospice/best supportive care** | M |  |  |  | 1 | 5 | 3 | 3 |  |  | 5.5 |  |
| 1. **Rating**: **A**-Usually appropriate; **M**-May be appropriate; **U**-Usually not appropriate 2. **Per the UCLA/RAND Appropriateness Method** - * Disagreement, i.e., the variation of the individual ratings from the median rating indicates panel disagreement on the final recommendation (see narrative text). Group median rating is set automatically to 5. | | | | | | | | | | | | |

**CASE 8 (a-e)** Progressive systemic and CNS NSCLC on a 3^rd^ generation TKI with a newly identified, targetable resistance mutation obtained by systemic biopsy. *In this variant, you will assume access to a novel TKI with variable CNS response rate and duration of response*.

Age 50

KPS 80

Extracranial disease progression on 3^rd^ generation TKI

# of brain metastases 12 asymptomatic metastases

Size of brain metastases all sub-centimeter,

Systemic therapy TKI targeted to new resistance mutation

**Variant 8a:**  Assume you have access to a novel TKI with CNS response rate <50% with a median CNS response duration of 6 months

| **Treatment** | **Rating Category** | **Final Tabulations** | | | | | | | | | **Group Median Rating** | **Disagree** |
| --- | --- | --- | --- | --- | --- | --- | --- | --- | --- | --- | --- | --- |
|  |  | **1** | **2** | **3** | **4** | **5** | **6** | **7** | **8** | **9** |  |  |
| **No Radiation, TKI alone** | M |  | 2 |  | 2 | 4 | 3 | 1 |  |  | 5 |  |
| **SRS to all metastases and TKI** | A |  |  |  |  | 1 | 2 | 7 | 1 | 1 | 7 |  |
| **SRS to selected metastases and TKI** | M* | 1 |  |  | 1 | 3 | 1 | 4 | 1 | 1 | 5* | X |
| **WBRT * then TKI** | M* |  |  |  |  | 2 | 3 | 6 | 1 |  | 5* | X |
| **WBRT* with concurrent TKI followed by TKI** | M | 1 |  | 1 | 3 | 4 | 2 | 1 |  |  | 5 |  |
| 1. **Rating**: **A**-Usually appropriate; **M**-May be appropriate; **U**-Usually not appropriate 2. **Per the UCLA/RAND Appropriateness Method** - * Disagreement, i.e., the variation of the individual ratings from the median rating indicates panel disagreement on the final recommendation (see narrative text). Group median rating is set automatically to 5. | | | | | | | | | | | | |

**Variant 8b:**  Assume you have access to a novel TKI with CNS response rate 50% with a median CNS response duration of 8 months

| **Treatment** | **Rating Category** | **Final Tabulations** | | | | | | | | | **Group Median Rating** | **Disagree** |
| --- | --- | --- | --- | --- | --- | --- | --- | --- | --- | --- | --- | --- |
|  |  | **1** | **2** | **3** | **4** | **5** | **6** | **7** | **8** | **9** |  |  |
| **No Radiation, TKI alone** | M | 1 |  | 1 |  | 6 | 3 | 1 |  |  | 5 |  |
| **SRS to all metastases and TKI** | A |  |  |  | 1 |  | 2 | 6 | 2 | 1 | 7 |  |
| **SRS to selected metastases and TKI** | M* | 1 |  |  |  | 2 | 3 | 5 |  | 1 | 5* | X |
| **WBRT * then TKI** | M* |  |  | 1 |  | 5 | 2 | 4 |  |  | 5* | X |
| **WBRT* with concurrent TKI followed by TKI** | M* |  |  | 3 |  | 5 | 2 | 2 |  |  | 5* | X |
| 1. **Rating**: **A**-Usually appropriate; **M**-May be appropriate; **U**-Usually not appropriate 2. **Per the UCLA/RAND Appropriateness Method** - * Disagreement, i.e., the variation of the individual ratings from the median rating indicates panel disagreement on the final recommendation (see narrative text). Group median rating is set automatically to 5. | | | | | | | | | | | | |

**Variant 8c:**  Assume you have access to a novel TKI with CNS response rate 65% with a median CNS response duration of 8 months

| **Treatment** | **Rating Category** | **Final Tabulations** | | | | | | | | | **Group Median Rating** | **Disagree** |
| --- | --- | --- | --- | --- | --- | --- | --- | --- | --- | --- | --- | --- |
|  |  | **1** | **2** | **3** | **4** | **5** | **6** | **7** | **8** | **9** |  |  |
| **No Radiation, TKI alone** | M* | 1 |  | 1 |  | 1 | 4 | 4 | 1 |  | 5* | X |
| **SRS to all metastases and TKI** | A |  |  |  |  | 2 | 1 | 6 | 2 | 1 | 7 |  |
| **SRS to selected metastases and TKI** | M* | 1 |  |  |  | 2 | 2 | 6 |  | 1 | 5* | X |
| **WBRT * then TKI** | M | 1 | 1 |  | 1 | 4 | 4 | 1 |  |  | 5 |  |
| **WBRT* with concurrent TKI followed by TKI** | M* |  |  | 2 | 2 | 5 | 1 | 2 |  |  | 5* | X |
| 1. **Rating**: **A**-Usually appropriate; **M**-May be appropriate; **U**-Usually not appropriate 2. **Per the UCLA/RAND Appropriateness Method** - * Disagreement, i.e., the variation of the individual ratings from the median rating indicates panel disagreement on the final recommendation (see narrative text). Group median rating is set automatically to 5. | | | | | | | | | | | | |

**Variant 8d:**  Assume you have access to a novel TKI with CNS response rate 80% with a median CNS response duration of 14 months.

| **Treatment** | **Rating Category** | **Final Tabulations** | | | | | | | | | **Group Median Rating** | **Disagree** |
| --- | --- | --- | --- | --- | --- | --- | --- | --- | --- | --- | --- | --- |
|  |  | **1** | **2** | **3** | **4** | **5** | **6** | **7** | **8** | **9** |  |  |
| **No Radiation, TKI alone** | A |  |  |  |  | 3 |  | 4 | 4 | 2 | 7 |  |
| **SRS to all metastases and TKI** | A |  |  |  | 2 | 1 |  | 6 | 2 | 1 | 7 |  |
| **SRS to selected metastases and TKI** | M* |  |  |  | 1 | 2 | 2 | 5 | 2 |  | 5* | X |
| **WBRT * then TKI** | M* |  |  | 2 | 5 | 1 | 2 | 2 |  |  | 5* | X |
| **WBRT* with concurrent TKI followed by TKI** | M* |  | 1 | 2 | 5 | 1 | 2 | 1 |  |  | 5* | X |
| 1. **Rating**: **A**-Usually appropriate; **M**-May be appropriate; **U**-Usually not appropriate 2. **Per the UCLA/RAND Appropriateness Method** - * Disagreement, i.e., the variation of the individual ratings from the median rating indicates panel disagreement on the final recommendation (see narrative text). Group median rating is set automatically to 5. | | | | | | | | | | | | |

**Variant 8e:** Assume you have access to a novel TKI with CNS response rate 95% with a median CNS response duration of 18 months.

| **Treatment** | **Rating Category** | **Final Tabulations** | | | | | | | | | **Group Median Rating** | **Disagree** |
| --- | --- | --- | --- | --- | --- | --- | --- | --- | --- | --- | --- | --- |
|  |  | **1** | **2** | **3** | **4** | **5** | **6** | **7** | **8** | **9** |  |  |
| **No Radiation, TKI alone** | A |  |  |  |  | 1 |  | 2 | 6 | 4 | 8 |  |
| **SRS to all metastases and TKI** | M* |  |  | 1 | 1 | 1 | 3 | 2 | 3 | 1 | 5* | X |
| **SRS to selected metastases and TKI** | M* |  |  |  | 1 | 4 | 2 | 3 | 1 | 1 | 5* | X |
| **WBRT * then TKI** | M* | 1 | 1 | 2 | 3 | 4 | 1 |  |  |  | 5* | X |
| **WBRT* with concurrent TKI followed by TKI** | M* | 1 | 2 | 4 | 2 | 2 | 1 |  |  |  | 5* | X |
| 1. **Rating**: **A**-Usually appropriate; **M**-May be appropriate; **U**-Usually not appropriate 2. **Per the UCLA/RAND Appropriateness Method** - * Disagreement, i.e., the variation of the individual ratings from the median rating indicates panel disagreement on the final recommendation (see narrative text). Group median rating is set automatically to 5. | | | | | | | | | | | | |
